# An Adaptive Support Vector Machine Optimized by an Improved Starfish Optimization Algorithm for Hyperspectral Image Classification

**DOI:** 10.3390/biomimetics11080574

**Published:** 2026-08-11

**Authors:** Yi Zhang, Changyi Feng, Yong Xu

**Affiliations:** College of Electrical and Computer Science, Jilin Jianzhu University, Changchun 130119, China; zhangyi@jlju.edu.cn (Y.Z.); fengchangyi@student.jlju.edu.cn (C.F.)

**Keywords:** hyperspectral image classification (HSIC), starfish optimization algorithm, support vector machine (SVM)

## Abstract

This study proposes an adaptive Support Vector Machine (SVM) classification method based on an enhanced Starfish Optimization Algorithm (SFOAE-SVM) for hyperspectral image (HSI) classification. HSI classification remains a critical challenge in remote sensing due to the high dimensionality of spectral features, spectral mixing, scarcity of labeled samples, and complex land-cover distributions. The SFOAE algorithm is used for global hyperparameter optimization of SVMs, accounting for the distributional characteristics of the target HSI data. The approach aims to improve search capability and reduce the likelihood of convergence to local optima by combining multi-dimensional topology-oriented expansion with global exploration. Experimental results demonstrate that SFOAE-SVM achieves competitive classification accuracy and stable performance compared with conventional SVM parameter selection strategies and other optimization-based methods across three benchmark hyperspectral remote-sensing datasets. These results indicate that the proposed method offers a promising optimization-assisted SVM framework for hyperspectral remote-sensing image classification.

## 1. Introduction

Hyperspectral imaging has emerged as a powerful technology in remote sensing, capable of producing high-dimensional data with extremely precise spectral resolution. A hyperspectral image (HSI) consists of a large number of consecutive spectral bands and incorporates abundant spectral features, which enable superior classification performance. HSI carries rich spectral information, characterized by hundreds of continuous, narrow spectral bands. This property has been widely leveraged in numerous fields, including precision agriculture [1], mineral analysis [2,3], urban planning [4], and military target detection [5]. HSI contains more spectral–spatial information, enabling the discrimination of ground objects and fine-grained classification compared with visible-light images. Currently, HSI classification is widely used in the remote sensing community. Effective classification results are of great significance for extracting ground object information and guaranteeing high robustness.

The highly coupled, high-dimensional nature of HSI image data also poses severe challenges for data analysis, despite HSI’s considerable advantages for ground object detection. They inevitably introduce substantial redundancy and lead to the curse of dimensionality, although hundreds of spectral bands provide abundant features for ground objects. Furthermore, phenomena such as “same object with different spectra” and “different objects with the same spectrum” caused by complex ground object environments, together with significant noise interference in actual remote sensing acquisition scenarios, greatly increase the difficulty of solving nonlinear decision problems in high-dimensional spectral space. How to obtain hyperspectral ground object classification results with high accuracy from highly coupled, highly redundant spectral–spatial data is a key challenge currently facing the remote sensing community.

Numerous hyperspectral image (HSI) classification methods have been developed to address these challenges. Most current techniques require iterative optimization. For instance, supervised learning methods such as Support Vector Machine (SVM) [6,7,8], K-Nearest Neighbors (KNN) [9], Multiple Linear Regression (MLR) [10], and Random Forest (RF) [11] require iterative hyperparameter tuning and model evaluation. Unsupervised clustering methods, including K-means [12,13] and Fuzzy C-means (FCM) [14,15], iteratively update cluster centers until convergence. Dimensionality reduction and embedding approaches, such as Principal Component Analysis (PCA) [16] and Non-negative Matrix Factorization (NMF) [17,18], also involve iterative computations to optimize projection matrices. Although the aforementioned methods are capable of learning nonlinear features and leveraging high-dimensional information, they still share a common limitation: they are relatively sensitive to initial hyperparameters and clustering centers, have limited capability in processing non-convex data, and are prone to interference. Conventional image classification methods have notable drawbacks: they yield poor classification performance and are highly sensitive to parameter variations. In response, deep learning-based methods have increasingly been adopted for HSI classification to address these limitations. Deep learning approaches offer powerful feature representation capabilities, enabling the automatic extraction of effective features within the target domain and substantially advancing image classification. When applied to HSI classification, convolutional networks, fully convolutional networks, and recurrent structures capture joint spectral–spatial features. Attention modules remove redundant features and emphasize discriminative subspaces, while deep matrix or tensor decomposition embeddings learn low-rank structures and enhance nonlinear representation. Representative models include Autoencoder (AE) [19], Recurrent Neural Network (RNN) [20], Convolutional Neural Network (CNN) [21,22], and Long Short-Term Memory (LSTM) [23]. These models are integrated end-to-end to adaptively model spectral domain information in hyperspectral images, thereby eliminating the need for complex manual feature selection. Despite the effectiveness of CNNs in extracting spatial–spectral features for HSI classification, they have inherent limitations. Due to locality and parameter sharing, CNNs cannot capture complex long-range dependencies. Conventional CNN architectures often fail to extract deep, explicit spectral information and are limited in their ability to address complex spatial and geographical variations. Beyond model structure, data-level constraints also present critical challenges. The HSI domain frequently encounters limited labeled samples, imbalanced class distributions, and variable acquisition environments. As a result, deep learning models that depend heavily on labeled data and computational resources often lack adaptability and generalization. Consequently, developing lightweight solutions that enhance interpretability and reduce reliance on labeled data holds significant academic and practical value for real-world hyperspectral remote sensing applications.

Traditional methodologies are confronted with the issue of over-segmentation, whereas neural network-based approaches require substantial data and computational resources. Consequently, there is a need for computationally efficient and accurate classification techniques. SVM remains a suitable choice for hyperspectral image classification due to its strong generalization ability and low structural risk. Intelligent optimization algorithms, recognized for their global optimization capacity, rapid convergence, and high precision, have been extensively applied and integrated with traditional approaches. Reference [24] introduces chaotic sequences into the Artificial Bee Colony (ABC) algorithm to optimize SVM for hyperspectral image classification, enhancing both parameter search efficiency and classification accuracy. Reference [25] demonstrates that SVM parameters and feature subsets can be simultaneously determined using the Ant Colony System (ACS). Reference [26] applies Ant Colony Optimization (ACO) to jointly perform hyperspectral feature selection and SVM parameter optimization, thereby improving the combined performance of feature selection and classification. Reference [27] presents a novel hyperspectral image classification method, HA-PSO-SVM, which integrates Harmonic Analysis (HA), Particle Swarm Optimization (PSO), and SVM. Reference [28] optimizes SVM using three intelligent optimization algorithms—PSO, Genetic Algorithm (GA), and Artificial Bee Colony (ABC)—to enhance overall SVM performance. Reference [29] increases classification accuracy by employing a Genetic Algorithm (GA) to automatically optimize key parameters of the classification model. Reference [30] combines the Zebra Optimization Algorithm (ZOA), Genetic Algorithm (GA), Particle Swarm Optimization (PSO), Grey Wolf Optimization (GWO) and SVM model for jujube variety classification. Reference [31] improves classification performance and stability by integrating a hyperspectral classification framework with Particle Swarm Optimization (PSO) to jointly optimize band selection and SVM parameters. Reference [32] proposes a novel hybrid optimization method (IDHCHO) based on the integration of the Cheetah Optimization Algorithm (COA) and the Hippopotamus Optimization Algorithm (HOA), which is applied to feature selection and prediction/anomaly detection of wind power systems and significantly reduces RMSE on multiple datasets. Reference [33] proposes MYIGWO with double mutation and a chaotic adaptive neighborhood, which conducts search via double mutation, direction correction, and chaotic enhancement and is applied to engineering problems and path planning. Reference [34] proposes an improved crayfish optimization algorithm based on multi-mode cooperative search and dynamic distribution perturbation, which is designed to enhance the search capability and convergence speed of COA. Reference [35] proposes a DOA-SVM method that employs the Dream Optimization Algorithm to optimize SVM for shale lithofacies prediction. Reference [36] proposes the IWOA-SVM, an improved whale optimization algorithm based on a hybrid strategy, which is developed to optimize the key parameters of SVM for solving the optimization problem of cable force in reinforced suspension bridges. Reference [37] proposes an improved chaotic adaptive whale optimization algorithm (CAWOA) to optimize the parameters of SVM, thereby improving the performance of SVM. These proposed and improved optimization algorithms have substantially promoted the development of intelligent optimization algorithms, improved their overall performance, and enabled their application to optimization problems in various fields.

This paper proposes an improved Starfish Optimization Algorithm with Enhancement (SFOAE), a bio-inspired intelligent optimization method that simulates three key behaviors of starfish to enable search and optimization. The proposed algorithm integrates several critical enhancement strategies across three core stages: novel strategies are introduced during population initialization, exploration, and exploitation, respectively, to improve overall performance. Specifically, a piecewise linear chaotic map based on ergodic theory is adopted to increase the diversity and high randomness of the initial population; an anomalous super-diffusion walk based on the Hurst exponent is incorporated in the exploration phase to enhance the algorithm’s long-distance exploration capability and its ability to escape from local optima; adaptive quantum tunneling noise mutation is introduced in both exploration and exploitation phases to accelerate the algorithm’s convergence speed; and finally, bimodal topological crossover based on environmental perception and external archiving is added to strengthen the algorithm’s global exploration capability. All these modifications are implemented and abstracted from physical or mathematical theories to develop a more efficient, robust, and adaptable computational model. The overall objective of the SFOAE-SVM hyperspectral image classification framework is to optimize the penalty parameter C and the RBF kernel parameter σ so as to achieve superior performance in terms of classification accuracy, precision, and computational efficiency.

Experimental results indicate that the proposed algorithm effectively addresses the challenges of high computational overhead and complex parameter tuning associated with traditional classification methods. Classification outcomes on three benchmark HSI datasets demonstrate that the proposed method outperforms previous approaches, with effective improvements in classification accuracy. The main contributions are summarized as follows:

1. Improved algorithm (SFOAE-SVM): Building on a thorough analysis of the original SFOA, targeted improvements are defined, and the starfish optimization algorithm is enhanced through four key steps. In accordance with the SVM framework, SFOAE-SVM is proposed for hyperspectral image classification, and the SFOAE-SVM algorithm’s flowchart is provided.
2. Validation of the improved algorithm: Experimental results on CEC2022 test functions are presented to verify the effectiveness of the improved algorithm. The performance of the proposed SFOAE is compared with both classical intelligent optimization algorithms and state-of-the-art methods. Quantitative analysis demonstrates that SFOAE exhibits superior stability and convergence capability compared with other methods.
3. Practicability and generality of the algorithm: The practicability and generality of the SFOAE-SVM algorithm are verified through its application to hyperspectral image classification. Validation on three benchmark HSI datasets and comparison with multiple existing hyperspectral classification methods confirm the superiority of the proposed algorithm.

## 2. Related Works

### 2.1. SVM

**Figure 1:**
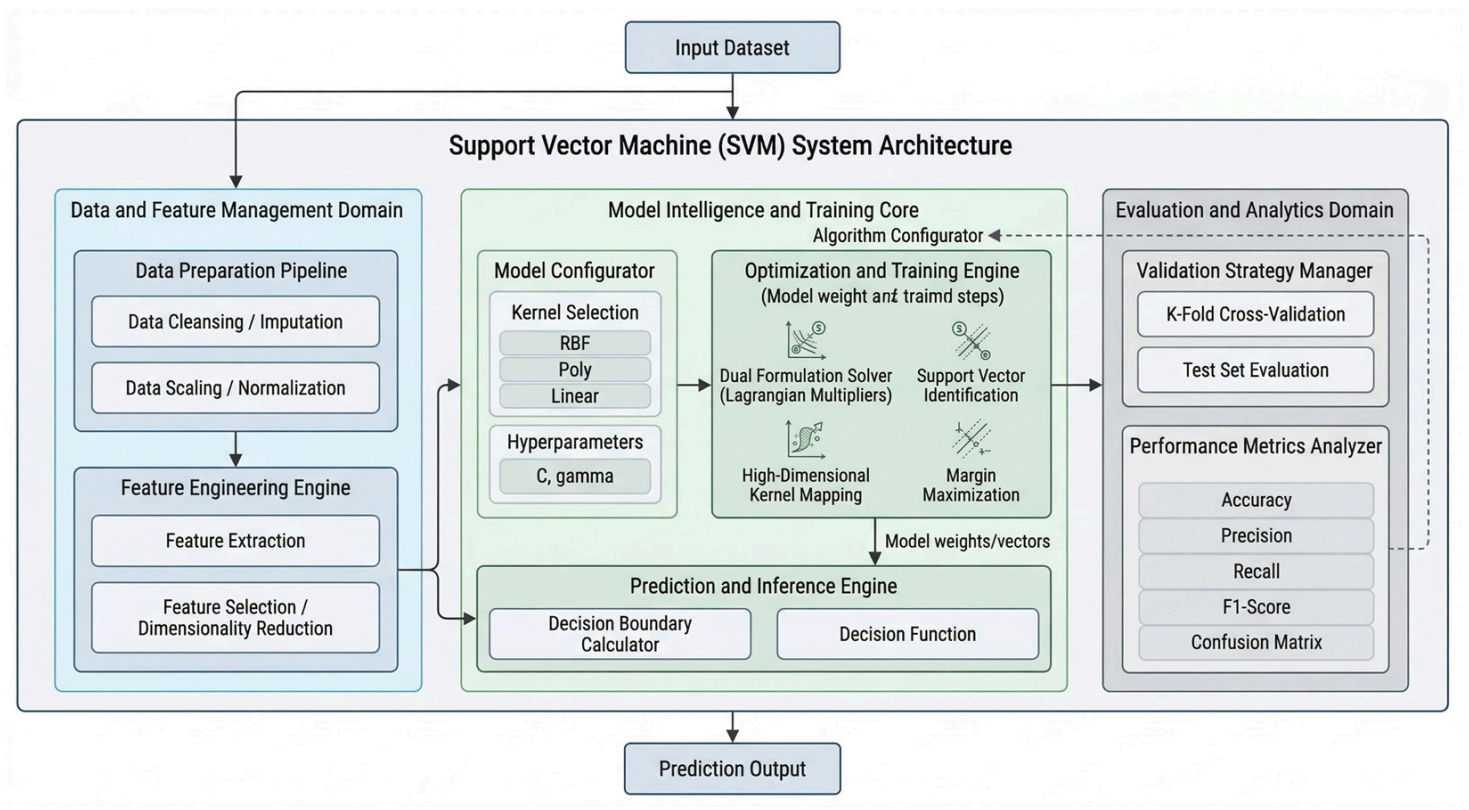
SVM structure diagram.

Support Vector Machine (SVM) [38] is one of the most widely used machine learning algorithms. The goal of SVM is to use hyperplanes to separate different classes. The performance of SVM is greatly affected by nonlinearly separable data. However, this problem can be solved by using kernel functions to transform the data from the input space to a new higher-dimensional space, where the transformed data can be easily separated. Selecting an appropriate kernel function and adjusting its parameters can help the SVM classifier create a linear decision through nonlinear transformation. The algorithm structure of SVM is shown in Figure 1.

Let the training sample set be ${\left\{\left(x_{i}y_{i}\right)\right\}}_{i=1}^{N},x_{i}\in R^{d},y_{i}\epsilon \left\{-1,+1\right\}$. For the case of linear separability, SVM will search for a hyperplane $w^{T}x+b=0$ in the feature space to maximize the distance between the sought hyperplane and the two types of samples to be classified, that is, the minimum distance from the samples to the hyperplane. By satisfying $y_{i}\left(w^{T}x_{i}+b\right)>1,\forall i=1,2,\ldots ,N$, it is possible to ensure that all samples are correctly classified. The margin width can be expressed as $\frac{2}{\left\|w\right\|}$. Therefore, the optimal classification hyperplane can be represented by Equation (1), which is a constrained optimization problem.

(1) $$\begin{array}{c} \underset{w,b}{\min}\frac{1}{2}{\left\|w\right\|}^{2}\\ s.t.\;\;y_{i}\left(w^{T}x_{i}+b\right)-1\geq 0,\;\;\forall i=1,2,\ldots ,N \end{array}$$

where $\underset{w,b}{\min}\frac{1}{2}{\left\|w\right\|}^{2}$ is the objective function and $y_{i}\left(w^{T}x_{i}+b\right)-1\geq 0$ is the constraint condition. This equation can be obtained by introducing Lagrange multiplier to obtain the Lagrangian function, as shown in Equation (2).

(2) $$L\left(w,b,\alpha \right)=\frac{1}{2}{\left\|w\right\|}^{2}-\sum_{i=1}^{N}\alpha_{i}\left(y_{i}\left(w^{T}x_{i}+b\right)-1\right)$$

(3) $$w=\sum_{i=1}^{N}\alpha_{i}y_{i}x_{i}$$

(4) $$\sum_{i=1}^{N}\alpha_{i}y_{i}=0$$

where $\alpha_{i}\geq 0$ represents Lagrange multipliers, and each Lagrange multiplier corresponds to a training mode, where the value of w,b,α can be calculated by taking the partial derivative of w,b and zeroing the derivative to obtain Equations (3) and (4). After solving, the optimal decision function can be obtained, as shown in Equation (5).

(5) $$f\left(x\right)=sgn\left(\sum_{i=1}^{N}\alpha_{i}y_{i}\left(x_{i}^{T}x\right)+b\right)$$

The data is often not linearly separable due to factors such as noise and mixed pixels in actual hyperspectral images. A relaxation variable $\xi_{i}\geq 0$ is introduced to allow some samples to be misclassified. The constraint condition then changes to $y_{i}\left(w^{T}x_{i}+b\right)-1+\xi_{i}\geq 0$. Thus, the optimization objective changes to that shown in Equation (6).

(6) $$\begin{array}{c} \underset{w,b,\xi }{\min}\frac{1}{2}{\left\|w\right\|}^{2}+C\sum_{i=1}^{N}\xi_{i}\\ s.t.\;\;y_{i}\left(w^{T}x_{i}+b\right)-1+\xi_{i}\geq 0,\;\;\forall i=1,2,\ldots ,N \end{array}$$

where C>0 is a penalty parameter to trade off the balance between margin maximization and misclassification minimization.

The SVM utilizes kernel techniques to map the original input space to a high-dimensional feature space through the mapping function $\phi :x\to \phi \left(x\right)$, enabling linear separability for nonlinearly separable data in this space since the spectral space distribution of most hyperspectral pixels is nonlinear. Thus, the optimization objective becomes as shown in Equation (7), and by introducing Lagrange multipliers, the Lagrangian function is obtained as shown in Equation (8). The values of w,b,α,μ,C can be calculated by taking the partial derivatives of w,b,ξ and setting the derivatives to zero, as shown in Equations (9)–(11). The final optimal decision function is shown as Equation (12).

(7) $$\begin{array}{c} \underset{w,b,\xi }{\min}\frac{1}{2}{\left\|w\right\|}^{2}+C\sum_{i=1}^{N}\xi_{i}\\ s.t.\;\;y_{i}\left(w^{T}\phi \left(x_{i}\right)+b\right)-1+\xi_{i}\geq 0,\;\;\;\;\forall i=1,2,\ldots ,N \end{array}$$

(8) $$\begin{array}{cc} L\left(w,b,\xi ,\alpha ,\mu \right)= & \frac{1}{2}{\left\|w\right\|}^{2}+C\sum_{i=1}^{N}\xi_{i}-\sum_{i=1}^{N}\alpha_{i}\left(y_{i}\left(w^{T}\phi \left(x_{i}\right)+b\right)-1+\xi_{i}\right)\\ & -\sum_{i=1}^{N}\mu_{i}\xi_{i} \end{array}$$

(9) $$w=\sum_{i=1}^{N}\alpha_{i}y_{i}\phi \left(x_{i}\right)$$

(10) $$\sum_{i=1}^{N}\alpha_{i}y_{i}=0$$

(11) $$C=\alpha_{i}+\mu_{i}$$

(12) $$f\left(x\right)=sgn\left(\sum_{i=1}^{N}\alpha_{i}y_{i}k\left(x_{i},x\right)+b\right)$$

where $\alpha_{i}\geq 0,\mu_{i}\geq 0$ represent the Lagrange multiplier, and $k\left(x_{i},x_{j}\right)={\phi \left(x_{i}\right)}^{T}\phi \left(x_{j}\right)$ is the kernel function, which is mainly used to map the sample space to the high-dimensional feature space. Common kernel functions include the polynomial kernel function, radial basis function (RBF), and sigmoid kernel function. In this study, the RBF kernel function is applied to the SVM classifier, as shown in Equation (13).

(13) $$k\left(x_{i},x_{j}\right)=exp\left(-\frac{{\left\|x_{i}-x_{j}\right\|}^{2}}{\sigma^{2}}\right)$$

where σ is a parameter of the kernel function that controls the degree of nonlinearity of the high-dimensional map.

The penalty parameter C and the RBF kernel function parameter σ are the main factors influencing the performance of the SVM classifier. Parameter C is used to balance the trade-off between maximizing the gap and minimizing the number of misclassifications. The value of the σ parameter affects the mapping of the input data to a higher-dimensional space in the RBF kernel function. This paper regards the penalty parameter C and the RBF kernel function parameter σ as black-box optimization variables to address the issues of high subjectivity, long processing time and low optimization accuracy in the traditional SVM parameter selection process to address the issues of high subjectivity. An improved intelligent optimization algorithm is used to jointly optimize in the continuous logarithmic space, obtaining near-optimal parameters to improve the classifier performance, and then applying the SVM method to process hyperspectral images by constructing a target function centered on them.

### 2.2. Starfish Optimization Algorithm

Zhong, C. et al. proposed the Starfish Optimization Algorithm (SFOA) [39] in 2024. This algorithm simulates the three key behaviors of starfish in nature to achieve search and optimization. The algorithm is specifically divided into four stages: population initialization, exploration, exploitation, and regeneration.

#### 2.2.1. Initialization Stage

The upper and lower bounds for the position of the starfish are randomly generated, which can be represented by matrix (14) during the initialization stage of SFOA.

(14) $$X={\left[\begin{array}{cccc} X_{11} & X_{12} & \cdots & X_{1D}\\ X_{21} & X_{22} & \cdots & X_{2D}\\ \vdots & \vdots & \ddots & \vdots \\ X_{N1} & X_{N2} & \cdots & X_{ND} \end{array}\right]}_{N\times D}$$

where X is the matrix holding the position of the starfish and is of size N×D, N is the population size, and D is the dimension of the design variable. At the initialization stage, the position of each starfish is expressed by Equation (15).

(15) $$X_{ij}=l_{j}+r\left(u_{j}-l_{j}\right),\;\;i=1,2,\ldots ,N,\;\;j=1,2,\ldots ,D$$

where $X_{ij}$ represents the j-th dimensional position of the i-th starfish, r represents a random number between (0, 1), and $u_{j}$ and $l_{j}$ are the upper and lower limits of the design variable in the j-th dimension.

#### 2.2.2. Exploration Stage

The behavior of starfish in the ocean was simulated during the exploration phase. The mode in which starfish simultaneously search using all five arms during their exploration was adopted. In the exploration phase of SFOA, the five-dimensional search mode with D>5 and the one-dimensional search mode with D≤5 from different optimization problems were combined. The threshold of the dimension was determined based on the five arms of the starfish.

It indicates that the problem has a complex solution space, so five arms of the starfish need to be used for the search if the dimension of the optimization problem is greater than 5 (D>5). The movement of the starfish should be guided by the best positions of all the starfish in the position update of the starfish. The search strategy for this stage is represented by Equation (16).

(16) $$\left\{\begin{array}{c} Y_{i,p}^{T}=X_{i,p}^{T}+a_{1}\left(X_{best,p}^{T}-X_{i,p}^{T}\right)\cos\theta ,\;\;\;r\leq 0.5\\ Y_{i,p}^{T}=X_{i,p}^{T}-a_{1}\left(X_{best,p}^{T}-X_{i,p}^{T}\right)\sin\theta ,\;\;\;r>0.5 \end{array}\right.$$

(17) $$a_{1}=\left(2r-1\right)\pi$$

(18) $$\theta =\frac{\pi }{2}\cdot \frac{T}{T_{max}}$$

where $Y_{i,p}^{T}$ and $X_{i,p}^{T}$ represent the new and current position of the starfish, respectively. $X_{best,p}^{T}$ represents the optimal position of the current starfish in the dimension. p is randomly selected from the five dimensions in the D dimensional space. The calculation formulas for $a_{1}$ and θ are given by Equations (17) and (18). Where $r\in \left(0,\;1\right)$, T is the current iteration, and $T_{max}$ is the maximum iteration. The sine and cosine terms indicate that the arms of the starfish will rotate left and right with equal probability for exploration. During the exploration phase, the $a_{1}$ in each formula is randomly generated, and θ varies with the iteration number $\theta \in \left[0,\;\pi /2\right]$.

A one-dimensional search mode is used to update the new position of the starfish in the exploration stage if the dimension of the optimization problem is no greater than 5 (D≤5). In this situation, the starfish will only use one arm to obtain new information. The search strategy at this stage is represented by Equation (19).

(19) $$Y_{i,p}^{T}=E_{t}X_{i,p}^{T}+A_{1}\left(X_{k_{1},p}^{T}-X_{i,p}^{T}\right)+A_{2}\left(X_{k_{2},p}^{T}-X_{i,p}^{T}\right)$$

(20) $$E_{t}=\frac{T_{max}-T}{T_{max}}\cos\theta$$

where $X_{k_{1},p}^{T}$ and $X_{k_{2},p}^{T}$ are the positions of two randomly selected starfish in dimension p, $A_{1}$ and $A_{2}$ are two random numbers within the range of (−1, 1), and p is the randomly selected two dimensions in the D dimensional space. $E_{t}$ represents the energy of the starfish, which is expressed by Equation (20). θ is calculated by Equation (18).

#### 2.2.3. Exploitation Stage

The development stage incorporates simulations of starfish predatory and regenerative behaviors to achieve precise optimization in the SFOA. Accordingly, SFOA implements two update strategies. The SFOA applies a parallel bidirectional search strategy that leverages both the optimal positions within the starfish population and the positions of other individuals to replicate the predatory phase. Initially, the distances between the optimal starfish and five randomly selected starfish are calculated. Two of these distances are then randomly chosen. The parallel bidirectional search strategy is subsequently used to update each starfish’s position. The calculation of distances is defined in Equation (21), and the update rule for the hunting behavior is provided in Equation (22).

(21) $$d_{m}=\left(X_{best}^{T}-X_{m_{p}}^{T}\right),m=1,\ldots ,\;5$$

(22) $$Y_{i}^{T}=X_{i}^{T}+r_{1}d_{m1}+r_{2}d_{m2}$$

In the calculation of distance Equation (21), $d_{m}$ is the distance between the optimal starfish position and the other five randomly selected starfish, while $m_{p}$ is the distance between the five randomly selected starfish. In the predation behavior Equation (22), $r_{1}$ and $r_{2}$ are random numbers within the range of (0, 1), and $d_{m1}$ and $d_{m2}$ are randomly selected from $d_{m}$.

#### 2.2.4. Regeneration Stage

The starfish move slowly and are thus vulnerable to attacks from other predators during the hunting process. The starfish may get injured and lose an arm to avoid being captured if a predator catches the starfish. Therefore, the regeneration stage of SFOA is only carried out in the last starfish of the population (i=N). Their movement speed is very slow because starfish need a long period of recovery during the regeneration process. The update rule for the regeneration stage is updated by Equation (23) to update the position.

(23) $$Y_{i}^{T}=exp\left(-T\times N/T_{max}\right)X_{i}^{T}$$

where T is the current iteration, $T_{max}$ is the maximum number of iterations, and N is the population size.

## 3. An Adaptive Support Vector Machine Hyperspectral Image Classification Method Based on the Improved Starfish Optimization Algorithm

The support vector machine is optimized by enhancing the SFOA to address its tendency to become trapped in local optima and its slow convergence. The population is initialized using piecewise linear chaotic mapping (PWLCM). During the exploration stage, an abnormal super-diffusion random walk strategy based on the Hurst index (ASDW) is incorporated. In both the exploitation and exploration stages, an adaptive quantum tunneling effect mutation mechanism (QTE) is applied. Additionally, within the exploration stage, an environment-aware bimodal topological crossover strategy with an external archive (Bi-JADE) is integrated. These improvements are designed to broaden the algorithm’s search space, improve accuracy, increase precision, and accelerate convergence.

The performance of SVM is highly dependent on the selection and configuration of penalty parameters and kernel function parameters. Currently, the three primary methods for optimizing SVMs include empirical selection [40], grid search [41,42], and gradient descent [43]. Empirical selection relies on repeated experimentation and experience, resulting in high randomness and difficulty in achieving a global optimum. Grid search can improve SVM classification accuracy, but it is computationally intensive, and its effectiveness depends on the grid’s granularity. Gradient descent enables autonomous parameter search but converges slowly, requires numerous iterations, and is time-consuming. In comparison, SVM parameter optimization using intelligent optimization algorithms is considered a promising alternative.

**Figure 2:**
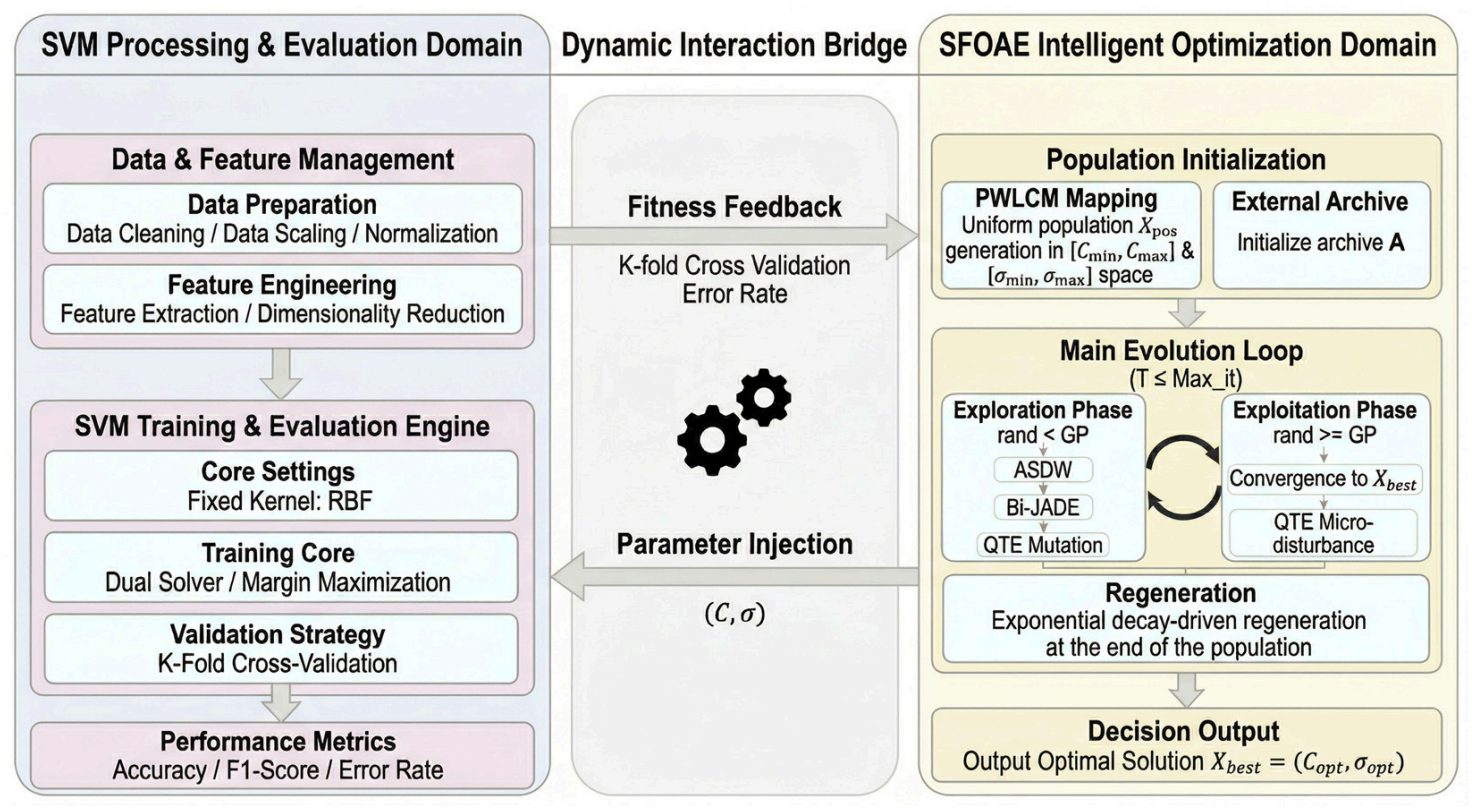
SFOAE-SVM structure diagram.

An improved SFOA algorithm, termed SFOAE-SVM, is proposed to optimize SVM parameters. In this approach, the penalty parameter C of SVM and the parameter σ of the radial basis function (RBF) kernel are treated as black-box optimization variables. A target function is constructed based on these variables, and the enhanced SFOA algorithm is employed to obtain near-optimal parameter solutions. This process facilitates hyperspectral image classification and enhances the overall classification accuracy. The structure of the proposed SFOAE-SVM algorithm is illustrated in Figure 2.

### 3.1. Initialization Mechanism of Piecewise Linear Chaotic Mapping Based on Ergodic Theory

**Figure 3:**
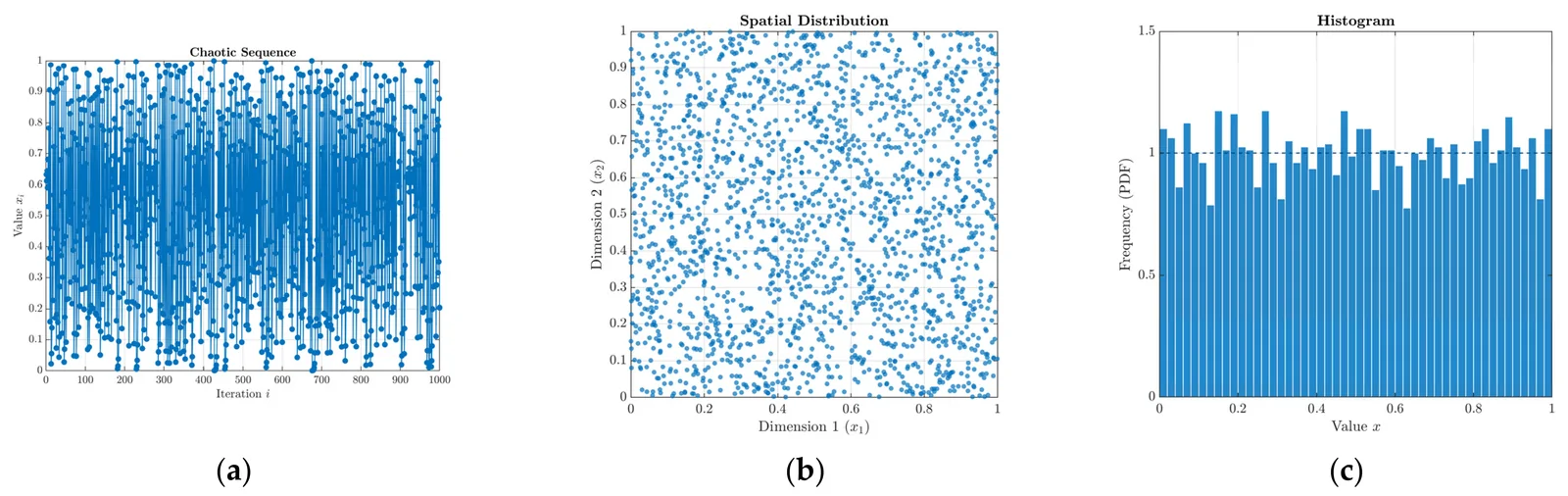
PWLCM: (**a**) chaotic sequence; (**b**) chaotic point plot; (**c**) chaotic histogram.

The selection of the initial population positions can directly affect the search efficiency, the global search capability, and the ability to avoid falling into local optimal solutions. An excellent initial overall distribution can help the algorithm cover the entire search space, increasing the chances of finding the global optimal solution. On the contrary, if the initial population positions are not chosen correctly, the population may overly concentrate in a certain area of the search space, thus falling into a local optimal solution and affecting the performance of the algorithm. The initial stage of the SFOA’s initialization process randomly generates boundaries, which cannot evenly cover the entire search space. This may result in missing the opportunity to find the global optimal solution and thus getting stuck in a local optimum. Therefore, in this paper, the initial population positions are generated using the piecewise linear chaotic mapping based on ergodic theory (PWLCM). PWLCM has an absolutely uniform probability density function ($f\left(x\right)=1$), which can ensure that the initial population achieves true ergodicity and absolute uniform distribution in the high-dimensional space, laying an excellent foundation for global optimization of the algorithm. The mathematical definition of PWLCM is shown in Equation (24).

(24) $$x_{i+1}=\left\{\begin{array}{c} \frac{x_{i}}{p},\;\;\;\;\;\;0\leq x_{i}\leq p\\ \frac{x_{i}-p}{0.5-p},\;\;\;\;\;\;p\leq x_{i}<0.5\\ \frac{1-x_{i}-p}{0.5-p},\;\;\;\;\;\;0.5\leq x_{i}<1-p\\ \frac{1-x_{i}}{p},\;\;\;\;\;\;1-p\leq x_{i}\leq 1 \end{array}\right.$$

where $x_{i}\in \left(0,\;1\right)$ represents the chaotic state value of the ith iteration. $p\in \left(0,\;0.5\right)$ is the control parameter of PWLCM (in this paper, it is set as p=0.4), which is used to control the bifurcation and dynamical folding behavior of the system. The sequence generated by PWLCM is shown in Figure 3.

Figure 3a presents the visualization result of PWLCM, while Figure 3b displays the chaotic sequence generated by PWLCM. Figure 3c illustrates the frequency distribution of each value, demonstrating that PWLCM produces uniformly distributed chaotic points within the specified range. The absence of obvious periodicity or repetitive structures, combined with strong nonlinearity and high randomness, indicates that PWLCM meets the requirements for generating the initial population.

### 3.2. Abnormal Super-Diffusion Random Walk Based on Hurst Index

The exploration formula in the exploration stage of SFOA largely determines the search performance of the algorithm, as it forms the basis for the algorithm’s global search. The traditional exploration formula of SFOA only uses random values to update the starfish position, resulting in an overly uniform step size distribution, which is prone to getting stuck in local optima and causing periodic direction updates. Therefore, the improved model incorporates the anomalous super-diffusion model from non-equilibrium statistical mechanics (ASDW). The ASDW strategy dynamically adjusts the Hurst index H, causing the search step size to exhibit heavy-tailed super-diffusion (H→1) in the early stages of iteration, enabling long-distance leap exploration to escape from local traps. In the later stages of iteration, the search step size transforms into Brownian motion (H→0.5), ensuring fine-scale local exploitation. Therefore, according to Equation $\left(16\right)$, an abnormal super-diffusion random walk strategy is introduced in the exploration stage. The improved search strategy is shown in Equation (25) when the dimension is greater than 5 (D>5).

(25) $$\left\{\begin{array}{c} Y_{i,p}^{T}=X_{i,p}^{T}+a_{1}\left(X_{best,p}^{T}-X_{i,p}^{T}\right)\cos\theta ,\;\;\;r\leq 0.5\\ Y_{i,p}^{T}=X_{i,p}^{T}-a_{1}\left(X_{best,p}^{T}-X_{i,p}^{T}\right)\sin\theta ,\;\;\;r>0.5 \end{array}\right.$$

(26) $$\theta =\frac{\pi }{2}\cdot \frac{T}{T_{max}}$$

(27) $$a_{1}=\left(2{Step}_{ASDW}-1\right)\pi$$

(28) $${Step}_{ASDW}=U\cdot {\left|V\right|}^{2H\left(t\right)-2},\;\;\;\;\;\;\;\;U,V∼N\left(0,\;1\right)$$

(29) $$H\left(t\right)=H_{max}-\left(H_{max}-H_{min}\right)\cdot \frac{T}{T_{max}}$$

If the dimension of the optimization problem is no greater than 5 (D≤5), then it is as shown in Equation (30).

(30) $$Y_{i,p}^{T}=E_{t}X_{i,p}^{T}+A_{1}\left(X_{k_{1},p}^{T}-X_{i,p}^{T}\right)+A_{2}\left(X_{k_{2},p}^{T}-X_{i,p}^{T}\right)$$

(31) $$E_{t}=\frac{T_{max}-T}{T_{max}}\cos\theta$$

(32) $$A_{1}=2{Step}_{ASDW}-1,\;\;A_{2}=2{Step}_{ASDW}-1$$

where $Y_{i,p}^{T}$ and $X_{i,p}^{T}$ represent the new and current positions of the starfish, respectively. $X_{best,p}^{T}$ represents the current optimal position of the starfish in dimension p, where p is randomly selected from the five dimensions in the D dimensional space. The values of $a_{1}$ and θ are calculated by Equations (26) and (27), respectively, where T is the current iteration, and $T_{max}$ represents the maximum iteration. ${Step}_{ASDW}$ is the abnormal super-diffusion random walk strategy calculated by Equation (28), where U and V are random numbers that follow the standard normal distribution, respectively. $H\left(t\right)$ is the Hirst index, which dynamically and nonlinearly decays with the number of iterations T, where the upper limit $H_{max}$ represents super-diffusion, and the lower limit $H_{min}$ represents normal random diffusion. $E_{t}$ represents the energy of the starfish, which is expressed by Equation (31). The values of $A_{1}$ and $A_{2}$ are calculated by Equation (32).

### 3.3. Adaptive Quantum Tunneling Effect Mutation

In the internal exploration of the algorithm, in order to overcome the problem of easily getting trapped in local optima in the complex solution space, this paper introduces the quantum tunneling effect (QTE) based on Schrödinger’s wave equation. Individuals trapped in local optima can produce sharp, peak-heavy-tailed mutations similar to those of the Laplace distribution by introducing quantum mechanics. It is possible to physically perturb the global optimal solution without affecting the population’s current position information, given the one-dimensional space-scale mapping matrix. This addition has accelerated the convergence speed of SFOA and enhanced its stability and accuracy. The location update formula is shown in Equation (33).

(33) $$P_{n}=X_{best}+\sigma Q_{noise}$$

(34) $$Q_{noise}=sgn\left(rand-0.5\right)\cdot \beta \left(T\right)\cdot \ln\frac{1}{u}$$

(35) $$\sigma =max\left(\eta_{min},\eta_{0}\left(1-\frac{T}{T_{max}}\right)\right)\cdot \left(ub-lb\right)$$

where $X_{best}$ is the current global optimal position vector, $u∼U\left(0,\;1\right)$ is a uniformly distributed random number, $\beta \left(T\right)$ is the quantum characteristic length that decays with iteration, and σ is the physical space scaling matrix. The double space contraction constraint is carried out by the initial step coefficient $\eta_{0}$ and the minimum coefficient $\eta_{min}$ to ensure that the tunneling distance is synchronized with the iterative process.

**Figure 4:**
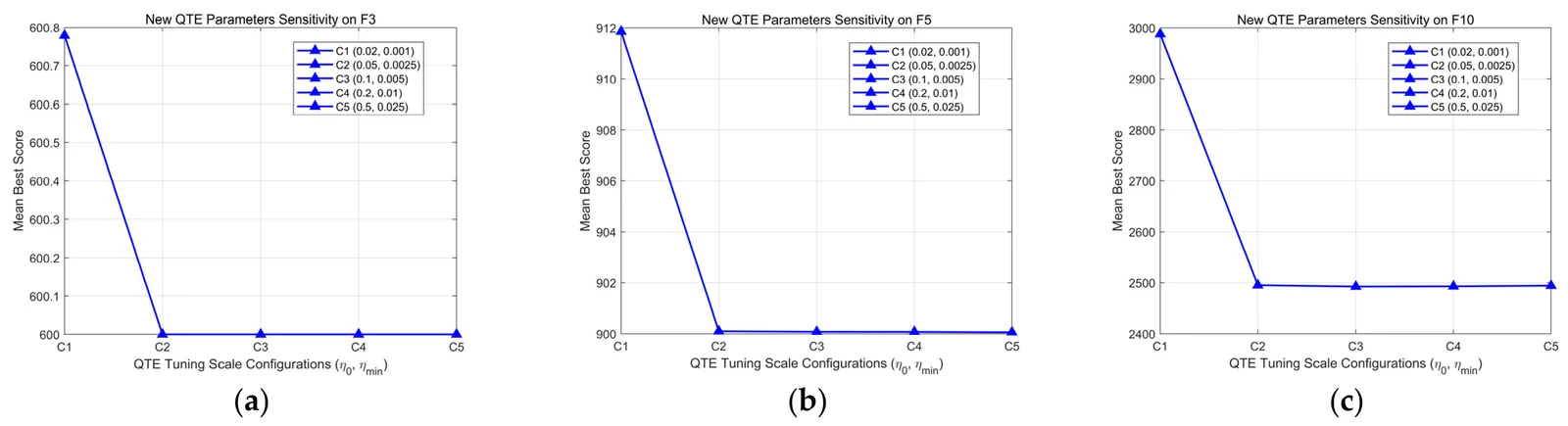
QTE parameter sensitivity analysis: (**a**) F3; (**b**) F5; (**c**) F10.

This paper conducts a sensitivity analysis of the parameters $\eta_{0}$ and $\eta_{min}$ using the test functions F3, F5, and F10 from the CEC2022 test suite to determine the initial values of the coefficients. The optimization results obtained from five different parameter combinations are shown in Figure 4. The selected initial coefficients $\eta_{0}=0.1$ and $\eta_{min}=0.005$ can yield better solutions in the optimization process and can be adopted as the initial coefficients for the improved strategy.

### 3.4. Environment-Aware Bimodal Topological Crossover Strategy with an External Archive

Individuals can use guidance from other excellent solutions within the group to conduct targeted expansion searches in the multi-dimensional topological space during the optimization process of intelligent optimization algorithms. However, traditional expansion searches are often limited by fixed topological structures and a single optimal solution orientation, which can easily cause the population to lose diversity in complex environments. Based on this logic, this paper introduces the bimodal topological crossover and the external archiving strategy (Bi-JADE).

This strategy first conducts targeted topological expansion around the known global-optimal elite region, guiding individuals towards high-quality areas. At the same time, an externally isolated physical archive is introduced along the topological expansion path to leverage the topological repulsion information from historically eliminated solutions, preventing the search process from getting stuck. The algorithm introduces an adaptive double-peak topological crossover during recombination to further increase the probability of obtaining new candidate solutions and to dynamically adapt to the complex coupling among variables. This deep integration of environmental perception and topological reorganization can not only significantly enhance the diversity of the search space but also effectively improve the convergence stability of the algorithm as it approaches the global extremum, thereby further improving the overall optimization performance. The mathematical formula for the environment-aware bimodal topological crossover strategy with an external archive is as follows:

(36) $${CR}_{i}=\left\{\begin{array}{c} N\left(\mu_{low},\sigma^{2}\right),\;\;\;\;\;\;if\;rand<0.5\\ N\left(\mu_{high},\sigma^{2}\right),\;\;\;\;\;\;else \end{array}\right.$$

(37) $$F_{i}=N\left(\mu_{F},\sigma_{F}^{2}\right)$$

Equation (36) represents adaptive bimodal parameter sampling. The crossover probability ${CR}_{i}$ of an individual is sampled using a bimodal Gaussian mixture model, and the scaling factor $F_{i}$ adopts an adaptive normal distribution where $\mu_{low}$ and $\mu_{high}$, respectively, control the tendency of low-dimensional recombination and high-dimensional recombination. $\mu_{F}$ and $\sigma_{F}$ are used to maintain the dynamic diversity of step sizes.

**Figure 5:**
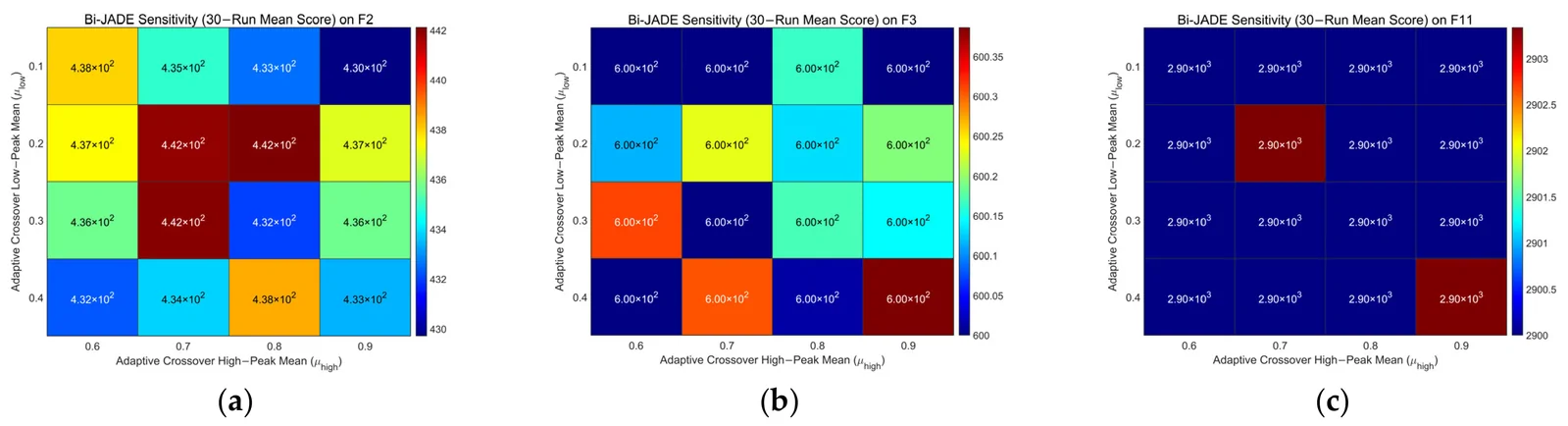
Bi-JADE parameter sensitivity analysis: (**a**) F2; (**b**) F3; (**c**) F11.

This paper conducts a sensitivity analysis of the parameters $\mu_{low}$ and $\mu_{high}$ using test functions F2, F3, and F11 from the CEC2022 test suite to determine the coefficient settings. The algorithm is executed 30 times, with the average results recorded for each group of candidate parameters, and the results are visualized as heatmaps to compare different parameter combinations. The results are shown in Figure 5: when the parameter combination is $\mu_{low}=0.1$ and $\mu_{high}=0.9$, the algorithm achieves better optimization performance. Therefore, this paper adopts $\mu_{low}=0.1$ and $\mu_{high}=0.9$ as the final coefficient settings.

(38) $$V_{i}=X_{i}+F_{i}\left(X_{pbest}-X_{i}\right)+F_{i}\left(X_{r1}-{\tilde{X}}_{r2}\right)$$

Equation (38) represents external archive-oriented differential mutation, where $X_{i}$ represents the current reference individual; $X_{pbest}$ is the target leader randomly selected from the elite solutions ranked in the top p% of the current population, responsible for providing the centripetal force for the directional expansion of the search; $X_{r1}$ is an individual randomly selected from the current population; and ${\tilde{X}}_{r2}$ is an individual randomly selected from the union of the current population P and the external archive A (i.e., P⋃A). The external archive A is specifically used to store the individuals eliminated in the previous generation, and it provides the external repulsive force for topological diversity to the current population through vector difference operation.

(39) $$U_{i,j}=\left\{\begin{array}{c} V_{i,j},\;\;\;\;\;\;if\;rand\leq {CR}_{i}\;or\;j=j_{rand}\\ X_{i,j},\;\;else \end{array}\right.$$

Equation (39) represents bimodal topological crossover enhancement. To further increase the generation probability of new solutions and the coverage of the search space, the topological guidance vector $V_{i}$ and the target vector $X_{i}$ are subjected to a multi-dimensional bimodal crossover operation to generate the final extended candidate solution $U_{i}$, where $j\in \left\{1,\;2,\ldots ,D\right\}$ represents the dimension index of the space, and $j_{rand}$ is a random integer within the range $\left[1,D\right]$, ensuring that the candidate solution strictly inherits the new topological direction of the directional expansion in at least one dimension within the manifold space.

**Figure 6:**
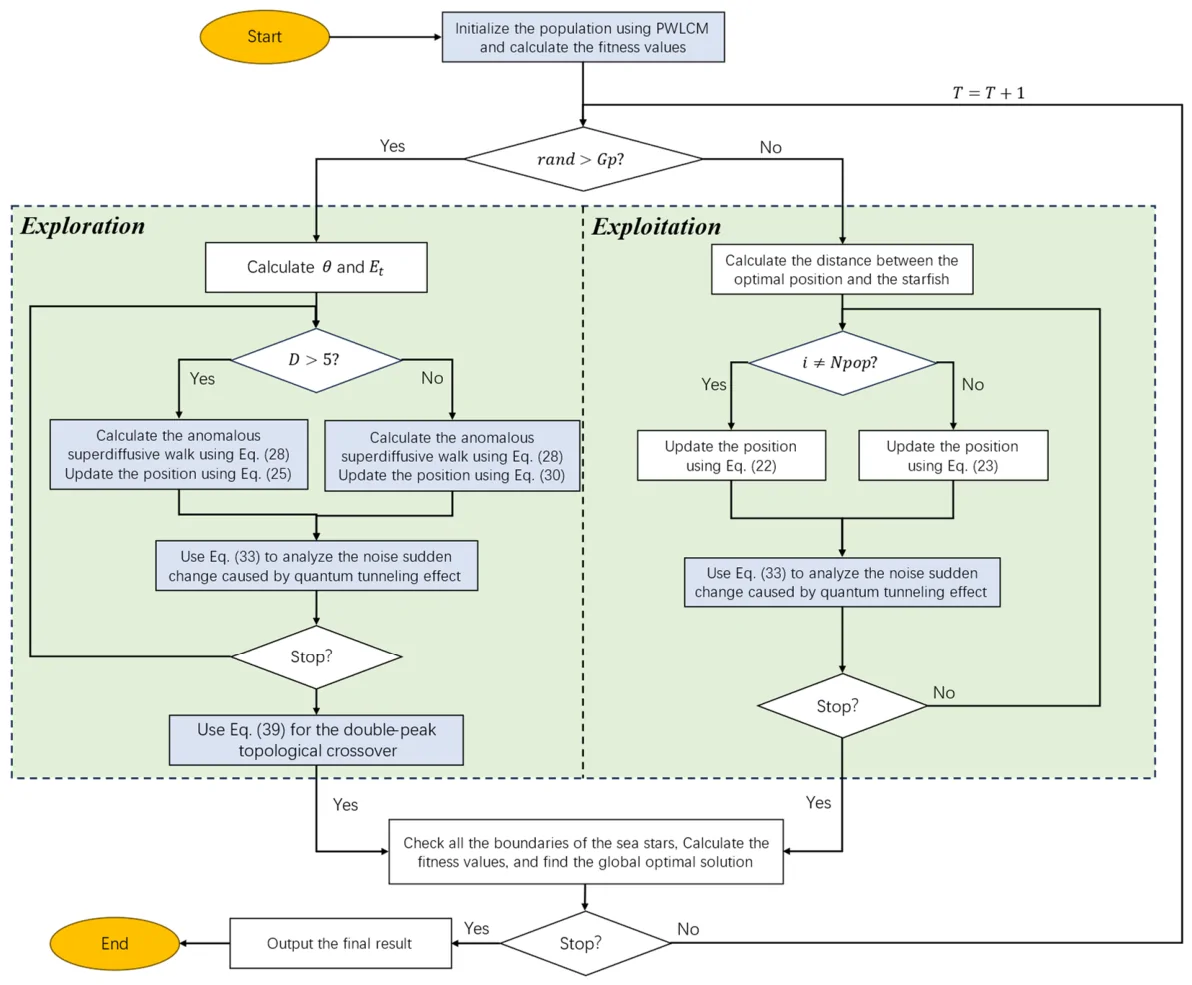
Flowchart of SFOAE.

The flowchart of the improved SFOAE algorithm based on the above steps is shown in Figure 6.

The pseudo-code of the improved SFOAE algorithm based on the above steps is as follows (Algorithm 1).
**Algorithm 1.** The pseudo-code of the SFOAE**Input:** Algorithmic parameters $\left(Npop,\;T_{max},\;D,Gp\right)$ **Output:** the global solution 1. Initialization stage 2. Initialize the position of the starfish using Equation (24) 3. Calculate the fitness value for each starfish 4. **While** $T<T_{max}$ **Do** 5.      **If** rand>Gp **Do** 6.          Calculate θ using Equation (26), and calculate $E_{t}$ using Equation (31) 7.          **For** Each starfish **Do** 8.              **If**
D>5
**Do** 9.                  Generate five random *p* dimensional vectors in *D* 10.                  **For**
*j* = 1:5 **Do** 11.                    Use Equation (28) to calculate ${Step}_{ASDW}$, and use Equation (27) to calculate $a_{1}$ 12.                    Update the positions of the starfish in the dimension using Equation (25) 13.                **End For** 14.                Check the boundary position 15.            **Else** 16.                Generate $A_{1}$ and $A_{2}$ using Equation (32) 17.                Select a random *p* dimensional one 18.                Update the position of the starfish in the dimension using Equation (30) 19.                Check the boundary position 20.            **End If** 21.            Use Equation (33) to perform adaptive quantum tunneling effect mutation 22.        **End For** 23.        Use Equation (39) to conduct the environment-aware bimodal topological crossover strategy with an external archive 24.    **Else** 25.        Calculate the optimal distance between the generated position and the starfish 26.        **For** Each starfish **Do** 27.            Generate a random number $r_{k}$ 28.            Update the position of the starfish according to Equation (22) 29.            **If** i=Npop **Do** 30.                Update the position using Equation (23) 31.            **End If** 32.            Check the boundary position 33.            Use Equation (33) to perform adaptive quantum tunneling effect mutation 34.        **End For** 35.    **End If** 36.    Calculate the fitness values of all starfish 37.    The global optimal solution is obtained at iteration step *T* 38.    T=T+1 39.  **End While** 40. Obtained the global solution

### 3.5. Comparison Between SFOA and SFOAE

This paper compares the SFOA and SFOAE from the perspective of their algorithmic mechanisms to further elaborate on the efficacy of the proposed algorithm improvement strategy. As illustrated in Table 1, the two algorithms exhibit significant differences in the initialization stage, exploration stage, mutation strategy, and population information interaction.

The original SFOA adopts a strategy of randomly generating distributions in the solution space during the initialization phase. However, this may cause the population to aggregate excessively in a certain region of the search space and fail to uniformly cover the entire search space, leading to local optima and impairing the algorithm’s final optimization result. The SFOAE introduces PWLCM and uses ergodic theory as the theoretical basis to initialize the population positions, ensuring that the population space is uniformly covered and laying a sound foundation for subsequent improvement strategies.

The formula in the exploration phase largely determines the search performance of the algorithm. As the foundation of global search, the original SFOA only uses random values for step size transformation during position update, which leads to excessively uniform step size distribution and makes the algorithm prone to falling into local optima. To address this deficiency, SFOA dynamically adjusts the search step size by introducing ASDW with the Hurst exponent as a guide, ensuring that the algorithm can explore the solution space more comprehensively.

The SFOA is prone to falling into local optima and converging prematurely. The SFOAE introduces the QTE strategy, which uses the identified global optimal individual to physically perturb the global optimal solution without altering the current population position information, thereby improving the algorithm’s ability to escape from local optima.

In the original SFOA, regardless of the exploration phase or the exploitation phase, the population position update is only related to the optimal position and the current position, which ignores information interaction among other population individuals and leads to a loss of algorithm diversity. Therefore, the SFOAE introduces the Bi-JADE strategy. By performing topological expansion around the global optimal solution and integrating the information of high-ranking elite solutions, it jointly provides topological diversity for the current population by utilizing the topological exclusion information of historically eliminated solutions so as to explore the solution space more comprehensively and enhance the optimization capability of the algorithm.

The SFOAE algorithm is improved in terms of the initialization phase, exploration phase, mutation strategy, and population information interaction, enabling the algorithm to escape from local optima and enhancing its optimization-seeking capability and convergence speed.

### 3.6. Time Complexity Analysis of Algorithm

Time complexity is a critical metric for evaluating algorithm performance. Let $T_{max}$ denote the maximum number of iterations, N the population size, D the dimensionality of decision variables, and $f\left(D\right)$ the computational complexity of a single fitness evaluation.

For SFOAE, the time complexity of the PWLCM initialization phase of the algorithm is $O\left(N\cdot \left(D+f\left(D\right)\right)\right)$; the time complexity of the newly added ASDW in the exploration phase is $O\left(N\cdot D\right)$; the time complexity of QTE is $O\left(N\cdot f\left(D\right)\right)$; and the time complexity of Bi-JADE is $O\left(N\cdot f\left(D\right)\right)$. The original baseline complexity is $O\left(N\cdot \left(D+f\left(D\right)\right)\right)$; in the exploitation phase, the original baseline complexity is $O\left(N\cdot f\left(D\right)\right)$, and the time complexity of the newly added QTE is $O\left(N\cdot f\left(D\right)\right)$. Therefore, the overall time complexity of SFOAE is $O\left(T_{max}\cdot N\cdot \left(D+f\left(D\right)\right)\right)$.

For the original SFOA, the time complexity of the algorithm’s initialization phase is $O\left(N\cdot \left(D+f\left(D\right)\right)\right)$, the time complexity of the exploration phase is $O\left(N\cdot \left(D+f\left(D\right)\right)\right)$, and the time complexity of the exploitation phase is $O\left(N\cdot f\left(D\right)\right)$. Therefore, the overall time complexity of SFOA is $O\left(T_{max}\cdot N\cdot \left(D+f\left(D\right)\right)\right)$.

By comparing the time complexity of SFOAE with that of the original SFOA, it can be observed that all the improved strategies introduced by SFOAE only increase the complexity in linear order, which merely adds a small amount of linear-order computational overhead, without introducing increases in complexity such as quadratic order or even cubic order. The overall time complexity of the algorithm remains at the level of $O\left(T_{max}\cdot N\cdot \left(D+f\left(D\right)\right)\right),$ which is consistent with the complexity of the original SFOA.

### 3.7. Adaptive Support Vector Machine Classification Method Based on SFOAE

The support vector machine classification method has strong discrimination ability, but it is highly sensitive to the penalty parameter C and the kernel function parameter σ. If the values of C or σ are improperly selected, it may lead to overfitting or underfitting, thereby weakening the subsequent classification performance and stability. In order to obtain the optimal parameter combination for achieving good classification, in Section 2.1 of this paper, the idea of using the RBF kernel function in SVM was adopted. The penalty parameter C and the RBF kernel function parameter σ were regarded as black-box optimization variables. By constructing a target function centered on them and using an improved intelligent optimization algorithm to jointly optimize in the continuous logarithmic domain space, near-optimal parameters are obtained to improve the performance of the classifier.

In the actual optimization process, optimization parameters are usually mapped to the base-2 logarithmic space for searching so as to achieve wide-area parameter coverage spanning multiple orders of magnitude. However, this logarithmic mapping mechanism causes optimization parameters to produce exponential drastic changes in the original physical space during continuous updating. Furthermore, affected by physical factors such as spectral overlap of ground objects, interference from mixed pixels and unbalanced sample size, the objective function constructed based on classification performance indicators exhibits high non-convexity and multi-modality. Such complex topological characteristics easily cause traditional optimization algorithms to fall into local optima, resulting in search stagnation, or generate disordered oscillation in parameter-sensitive regions.

To address this low-dimensional but highly nonlinear logarithmic parameter optimization problem, this paper introduces the SFOA and its enhanced variant SFOAE for global hyperparameter optimization. The unique multi-arm cooperative predation mechanism of SFOA endows the algorithm with excellent multi-modal dynamic search characteristics, enabling the realization of dynamic balance between global exploration and local exploitation in the two-dimensional logarithmic topological space. Its multi-arm parallel updating mechanism enables the algorithm to span exponential scale ranges and quickly locate potential global optimal solution regions, while the adaptive dynamic adjustment mechanism ensures that the algorithm can carry out high-precision local exploitation with fine step size when approaching the narrow optimal parameter distribution interval. This algorithmic structure can efficiently explore the non-convex multi-modal surface formed by random fluctuations in the fitness of hyperspectral classification, and exhibits extremely high optimization efficiency, convergence stability and adaptability to hyperspectral tasks in the two-dimensional hyperparameter space.

Let the position coordinate vector of the optimization-seeking individual in the SFOAE be $z={\left[z_{1},z_{2}\right]}^{T}\in R^{2}$. The logarithmic mapping operator for the two optimization parameters is defined as shown in Equation (40), and the boundary constraint space Ω for this joint optimization is defined by Equation (41). In this paper, the boundaries are specified as $z_{1}\in \left[-2,3\right],z_{2}\in \left[-2,2\right]$.

(40) $$C\left(z_{1}\right)=10^{z_{1}},\sigma \left(z_{2}\right)=10^{z_{2}}$$

(41) $$\Omega =\left\{z={\left[z_{1},z_{2}\right]}^{T}|z_{1}\in \left[z_{1,min},z_{1,max}\right],z_{2}\in \left[z_{2,min},z_{2,max}\right]\right\}$$

Let the sample set randomly drawn from the hyperspectral dataset be $D_{tr}={\left\{\left(x_{i},y_{i}\right)\right\}}_{i=1}^{N_{tr}}$. A K-fold partitioning operator is constructed to randomly divide $D_{tr}$ into K mutually disjoint subsets of equal size, satisfying $D_{tr}={⋃}_{k=1}^{K}D_{k}$ and $D_{k}\cap D_{j}=\emptyset \left(\forall k\neq j\right)$. For the k-th fold validation, the in-fold validation set is defined as $D_{va}^{\left(k\right)}=D_{k}$, and the in-fold training set is defined as $D_{tr}^{\left(k\right)}=D_{tr}\setminus D_{k}$. All in-fold data are transformed using the mean $\mu_{k}$ and standard deviation $s_{k}$ calculated from the in-fold training set $D_{tr}^{\left(k\right)}$, as shown in Equation (42).

(42) $${\tilde{x}}_{i}=\frac{s_{i}-\mu_{k}}{s_{k}^{*}}\left(\forall i\in D_{tr}^{\left(k\right)}\cup D_{va}^{\left(k\right)}\right)$$

To ensure the objectivity of classification accuracy evaluation and prevent data leakage and overfitting, the training dataset and validation dataset adopted in this paper are completely independent. Specifically, this study randomly extracts 10% of the samples to construct the training set, and the remaining 90% of samples, which are independent of the training samples, are used as the test set. Meanwhile, during the iterative training of SFOAE, the algorithm adopts 5-fold cross-validation with k=5 to calculate the objective function.

The standardized within-fold training set is input into the Support Vector Machine (SVM) based on Radial Basis Function (RBF) kernel for training, and its black-box training mapping process can be expressed by Equation (43).

(43) $$F_{k}=\Psi \left({\tilde{D}}_{tr}^{\left(k\right)};C,\sigma \right)$$

In this formulation, $F_{k}$ denotes the k-th generated multi-class decision model, and $\Psi \left(\cdot \right)$ represents the instantiated training operator for SVM.

The global objective function $fobj\left(z\right)$ of the SFOAE algorithm is defined as shown in Equation (44).

(44) $$\underset{z\in \Omega }{\min}fobj\left(z\right)=\frac{1}{K}\sum_{k=1}^{K}L_{k}\left(10^{z_{1}},10^{z_{2}}\right)\;s.t.L_{k}\left(C,\sigma \right)=\frac{1}{\left|D_{va}^{\left(k\right)}\right|}\sum_{i\in D_{va}^{\left(k\right)}}I\left(F_{k}\left({\tilde{x}}_{i}\right)\neq y_{i}\right)$$

Therein, $I\left(\cdot \right)$ denotes the indicator function. After the iteration terminates, the optimal solution $z^{*}$ output by the algorithm is restored via inverse mapping to obtain the near-optimal parameters, which are expressed as $C_{best}=10^{z_{1}^{*}}$ and $\sigma_{best}=10^{z_{2}^{*}}$.

The combination of the support vector machine classification method and SFOAE can avoid the drawbacks of traditional SVM, such as the difficulty in manually setting parameters. It can effectively find the optimal combination of solutions, addressing the problem of poor classification accuracy that occurs when using support vector machines alone. The pseudo-code of the proposed SFOAE-SVM algorithm is as follows (Algorithm 2).
**Algorithm 2.** The pseudo-code of the SFOAE-SVMInput: Original image Output: Classification result image 1. Image loading 2. Image preprocessing 3. Select 10% of the data as the training set 4. Define the population size, the number of iterations, the dimension, the upper bound, and the lower bound 5. Formulate the objective function 6. Start the SFOAE iterative loop 7. Algorithm 1 8. Obtain the penalty parameter *C* and the kernel function parameter σ 9. Carry out classification training using SVM 10. Obtain the classified result image

## 4. Experimental Results and Analysis

### 4.1. Experimental Environment

This section aims to evaluate the effectiveness and practicability of the improved algorithm by comparing it with other intelligent optimization algorithms. All experiments were conducted on a computer equipped with the Windows 10 operating system, Intel (R) Core (TM) i7-9750H 2.60 GHz CPU and 16 GB memory. The simulation experimental platform used was MATLAB R2024a.

### 4.2. Test Function

This paper conducts a comparative experiment using the CEC2022 test function [44]. The optimization function test set of CEC2022 consists of 12 single-objective test functions with boundary constraints, namely: unimodal function (F1), multi-modal functions (F2–F5), mixed functions (F6–F8), and combined functions (F9–F12). It can test the convergence performance of the algorithm as well as the balance ability between the global exploration and local development of the decision space. Therefore, it can effectively evaluate the performance of the algorithm and is one of the most widely used and classic test sets. Furthermore, the test functions in CEC2022 include mixed functions and combined functions, covering non-convex, multi-modal and high-dimensional scenarios. They are consistent with the joint optimization objective of the penalty parameter C and the kernel function parameter σ in SVM hyperspectral image classification and are suitable for this parameter optimization task. All the test functions in the CEC2022 test set are designed to solve minimization problems. $F_{i}^{*}$ is the optimal value, and D represents the dimension, as shown in Table 2.

Although the joint parameter optimization of SVM-based hyperspectral image classification only involves the penalty parameter C and kernel function parameter σ, it is still necessary to introduce the high-dimensional CEC2022 test suite to evaluate the improved algorithm. Due to the interference of noise and mixed pixels in HSI, the two-dimensional cross-validation constructed during SVM parameter optimization will exhibit a high-dimensional multi-modal spatial structure, which makes the complexity of the solution space similar to that of some high-dimensional multi-modal functions in CEC2022. Meanwhile, adopting the CEC2022 test suite to validate the improved algorithm is also intended to test the optimization capability of the algorithm in higher-dimensional complex spaces and increase the optimization redundancy of the algorithm so that it can better cope with the parameter optimization task in SVM-based HSI classification.

### 4.3. Performance Comparison Analysis

The improved SFOAE in this paper was compared with several classic intelligent optimization algorithms from recent years: Goose Algorithm (GOOSE) [45], Harris Hawks Optimizer (HHO) [46], Whale Optimization Algorithm (WOA) [47], Golden Jackal Optimization (GJO) [48], Sand Cat Swarm Optimization (SCSO) [49], Osprey Optimization Algorithm (OOA) [50], and Subtraction-Average-Based Optimizer (SABO) [51]. At the same time, it was compared with the original SFOA algorithm before the improvement. The overall population Npop for each optimization algorithm is set to 60, and the maximum number of iterations $T_{max}$ is set to 500. To reduce errors and ensure the accuracy of the experiments, each optimization algorithm is run independently 30 times, and the final average value is taken.

Table 3 presents the performance of various optimization algorithms on CECC2022, reporting minimum, average, and worst values. With the exception of test functions F8 and F12, SFOAE achieved optimal results across all statistical measures. For F8, SFOAE did not obtain the lowest worst value; however, its worst value was only marginally worse than SFOA’s, while its minimum and average values surpassed those of all other tested algorithms and the original algorithm. This outcome demonstrates that SFOAE can effectively exploit the search space to achieve optimal solutions. On F12, SFOA attained the best minimum and worst values, ranking second in average value. Although SFOAE exhibited a slight disadvantage in the combination function, it still effectively avoided local convergence by integrating improvement strategies, thereby identifying the optimal value. Compared to the original SFOA, SFOAE demonstrates enhanced optimization capability and satisfactory global optimization performance.

**Figure 7:**
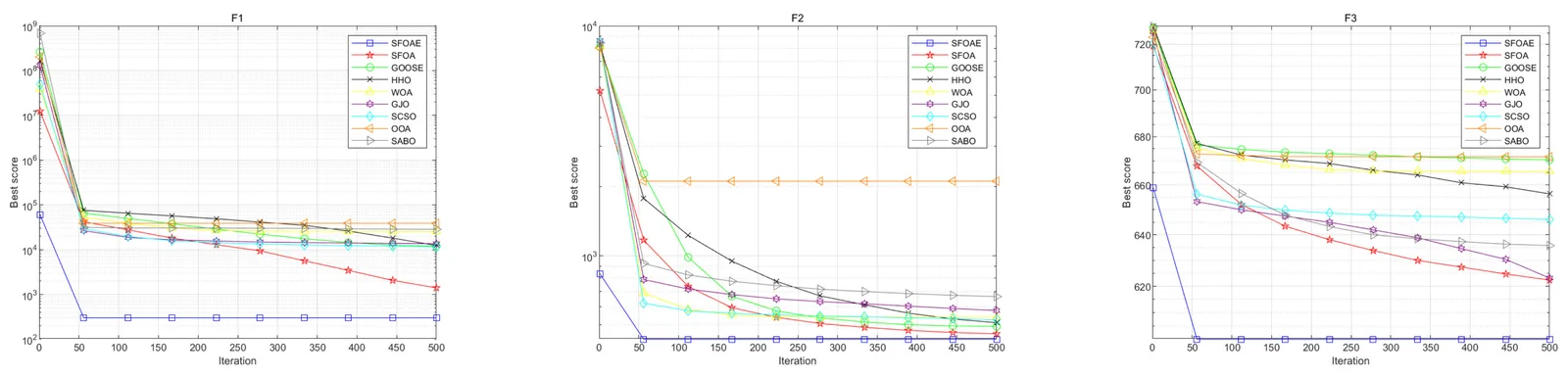

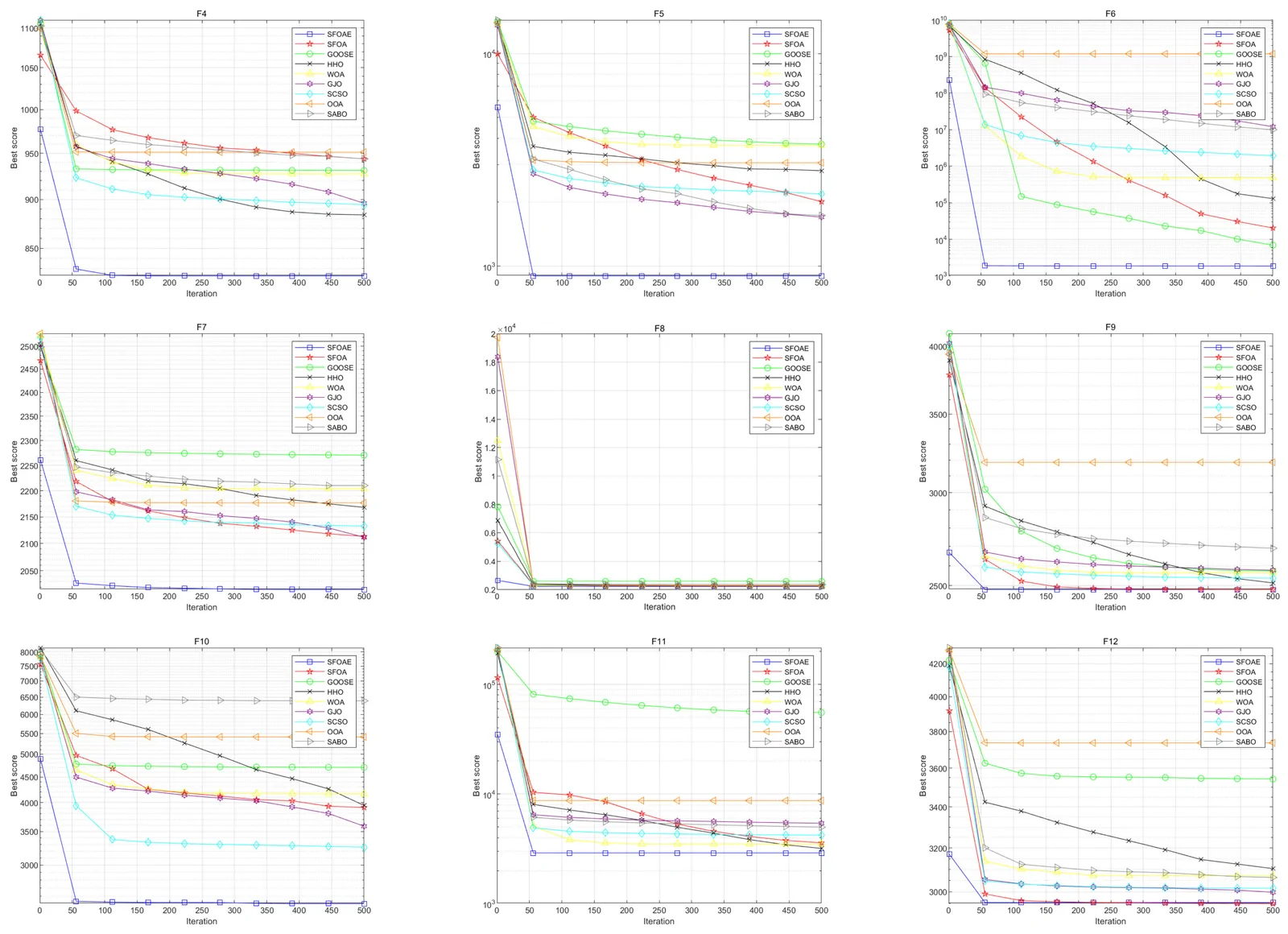
Comparison of the iterative curves of each algorithm on the CEC2022 test set.

To facilitate an intuitive comparison, an iterative curve was plotted to further verify the optimization performance of the proposed SFOAE. The convergence curves corresponding to the 12 test functions are shown in Figure 7.

Analysis of the iterative curves for all 12 functions in CEC2022 demonstrates that the improved SFOAE algorithm significantly outperforms the original SFOA and other comparison algorithms in initial solution quality, convergence speed, and final optimization accuracy. During early iterations, the uniform traversal property of PWLCM enables SFOAE to achieve a substantially lower initial fitness value than competing algorithms, effectively mitigating the risk of initial population aggregation. Most SFOAE functions display a rapid, cliff-like decline in fitness during early evolution. The integration of abnormal super-diffusion random walk and quantum tunneling mutations enhances the algorithm’s ability to escape local optima, allowing it to rapidly identify the global optimum region with relatively few evaluations. In later iterations, particularly when addressing complex, multi-modal functions, most comparison algorithms exhibit premature convergence and stagnation, whereas SFOAE maintains consistent progress. The algorithm’s local fine-grained exploitation and capacity to escape local optima confirm the effectiveness of the bimodal topological crossover and external archive mechanisms. Tracing the convergence trajectories on the CEC2022 test set indicates that SFOAE achieves a dynamic balance between global exploration and local exploitation, resulting in stable optimization performance across diverse and complex test environments.

**Figure 8:**
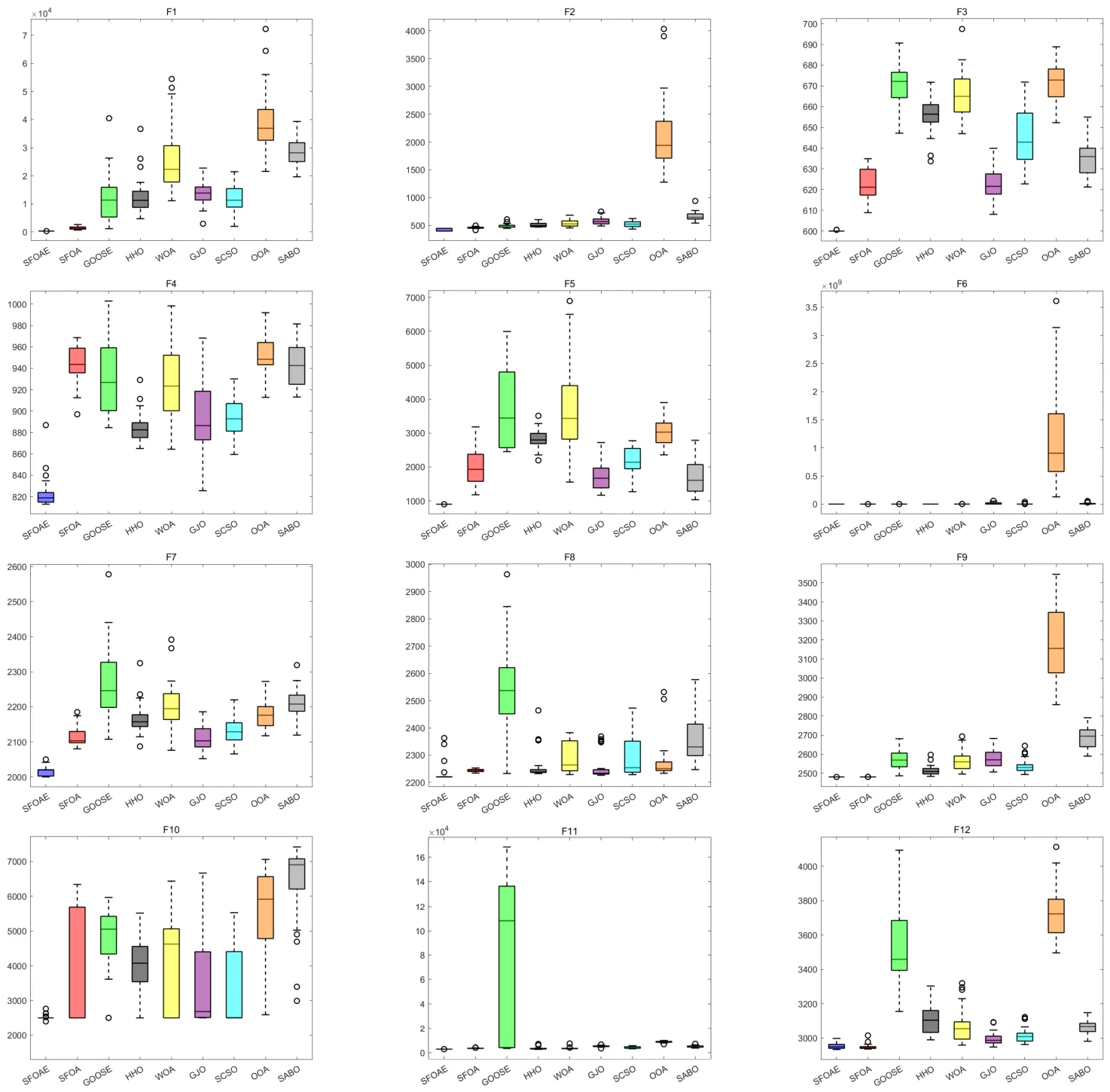
Comparison of box plots of each algorithm on the CEC2022 test set.

In this experiment, box plots were generated from the average optimization results of 30 independent runs for each test function using SFOAE and other optimization algorithms. The box plots for the 12 functions are presented in Figure 8.

The box plot [52] illustrates the distribution and dispersion of the 30 statistical results for each algorithm, thereby reflecting the stability of each algorithm’s performance during optimization of the IEEE CEC2022 test functions. A narrower frame range indicates that the algorithm’s values are more consistent across multiple runs, suggesting greater stability and reliability. A lower median value corresponds to higher optimization accuracy. According to the box plot results, with the exception of SFOAE in F12, the SFOAE box plots for all other functions are shorter, with median values lower than those of the other comparison algorithms and optimal values also lower. Consequently, SFOAE achieves superior optimization results across multiple runs, exhibits more stable performance, and can be considered a more reliable optimization algorithm.

### 4.4. Wilcoxon Rank-Sum Test

This study adopts the Wilcoxon Rank-Sum Test to conduct statistical analysis on the results obtained after 30 independent runs of all involved algorithms, with the aim of evaluating the performance difference between the SFOAE algorithm and the comparative algorithms. The difference between two algorithms is identified by the significance criterion of p<0.05, and comparison results with no significant advantage are highlighted in bold. The statistical results are presented in Table 4. For the first 11 test functions, the *p*-values between SFOAE and all other algorithms are all lower than 0.05, which indicates that the performance improvement of SFOAE is effective and statistically significant. Although only a minor improvement over the original SFOA is observed in the comparison on the F12 test function, this result still verifies that the proposed improvement strategy functions effectively. The results obtained via the rank-sum test show that the improvement strategy of SFOAE achieves a significant performance gain compared with the original SFOA, and the performance advantage of SFOAE is also confirmed in comparisons with other algorithms. This demonstrates that the improved SFOAE proposed in this paper is statistically effective and feasible.

### 4.5. Ablation Experiment

To verify the effectiveness of the proposed strategy, this paper conducts ablation experiments on the algorithm. By integrating different strategies individually into the original SFOA, four distinct algorithms are constructed. Among them, SFOAE_PWLCM only incorporates the PWLCM initialization mechanism; SFOAE_ASDW only includes the ASDW search mechanism; SFOAE_QTE only contains the QTE mutation mechanism; and SFOAE_BiJADE only comprises the BiJADE population information interaction mechanism.

Using the CEC2022 test suite, six representative test functions, namely F1, F2, F5, F6, F7 and F9, are selected to verify the effectiveness of the proposed strategy. To reduce experimental errors and confirm the reliability of experimental results, each algorithm is independently run 30 times to obtain the average value in experiments, with the population size set to 60 and the maximum number of iterations set to 500.

**Figure 9:**
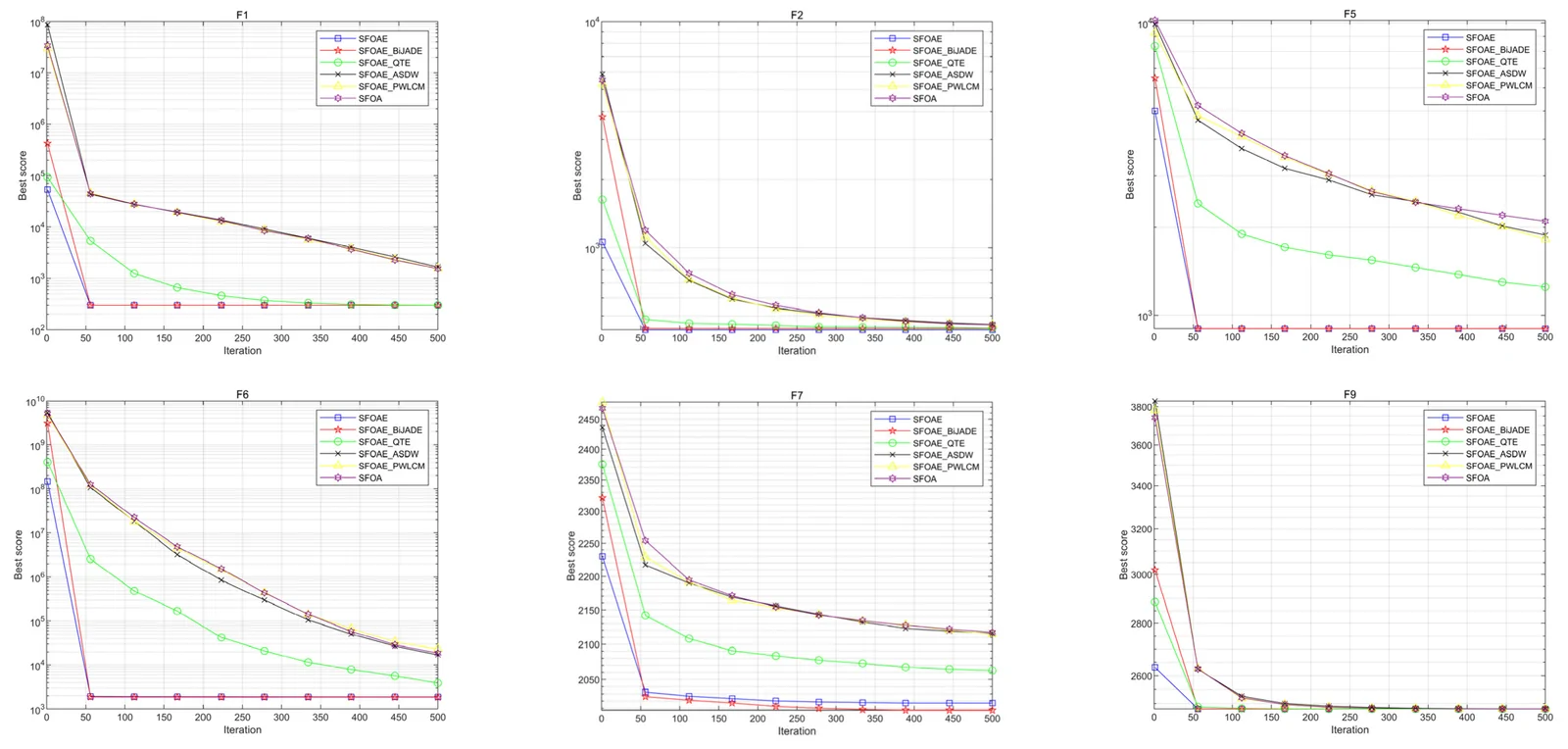
Ablation experiment convergence curve.

The results of the ablation experiment are shown in Figure 9. By observing the convergence curves of the six test functions, it can be observed that the QTE strategy and the BiJADE strategy produce remarkably significant effects. In the early iteration stage, both strategies exhibit a faster convergence rate; in the late iteration stage, both strategies enable the algorithm to jump out of local optima and obtain superior optimization results. Although the PWLCM strategy and the ASDW strategy do not bring a sufficiently obvious improvement to the original algorithm, after the population is reconstructed by PWLCM and the search step size is increased by ASDW, only a slight improvement in the convergence rate and global optimization capability of the algorithm can be observed. However, by comparing the other two strategies with SFOAE, it can be found that the population position reconstructed by the PWLCM strategy and the step size increased by the ASDW strategy still exert a partial effect on the overall algorithm when combined. The organic combination of the four strategies constitutes the excellent global optimization capability of SFOAE.

The statistical results of the ablation experiment are shown in Table 5. By comparing the test results of the single-strategy models, both the PWLCM strategy and the ASDW strategy achieve varying degrees of improvement over the original SFOA. This indicates that the population initialization strategy based on PWLCM enhances the diversity of the initial population and establishes a favorable population foundation for the implementation of subsequent strategies. However, the introduction of population diversity may also expose the imbalance problem of the original algorithm in the search process, thus leading to performance degradation. In contrast, the ASDW strategy improves the dynamic balance between local exploitation and global exploration.

The introduction of the QTE strategy and the BiJADE strategy brings more significant performance improvement to SFOA. In particular, the experimental result of the BiJADE strategy is very close to the overall improvement result of SFOAE. Although each single strategy improves the performance of SFOA to varying degrees, no single strategy can guarantee to obtain the optimal solution on all test functions. However, the results of SFOAE prove that the combined application of the four strategies can achieve results that fully outperform the original SFOA, which verifies that the four strategies have good complementary and synergistic capabilities, thus ensuring the excellent performance of the final proposed SFOAE.

## 5. Application Experiment of SFOAE-SVM in Hyperspectral Images

The previous section addressed the effectiveness and stability of the proposed algorithm. The experiments were performed using hyperspectral images to further validate its practicality. Advances in imaging spectroscopy technology have enabled simultaneous improvements in spectral and spatial resolution, producing high-spectral-resolution images with hundreds of narrow bands. In contrast to conventional multispectral RGB images, hyperspectral data offer enhanced spectral discrimination but also introduce significant challenges in modeling and computation. Hyperspectral images frequently exhibit nonlinear characteristics, and individual pixels often represent mixtures of multiple landforms, resulting in blurred boundaries. Additionally, the spatial neighborhood structure is complex, the range of target scales is broad, and small targets and edge regions are more susceptible to misclassification. These factors contribute to increased computational demands, extended processing times, and reduced classification accuracy in hyperspectral image analysis.

Hyperspectral image classification techniques are generally divided into machine learning and deep learning approaches. Machine learning methods include K Nearest Neighbors (KNN) [9], Random Forest (RF) [11], Multiple Linear Regression (MLR) [10], and Support Vector Machine (SVM) [6,7,8]. Deep learning methods primarily utilize Convolutional Neural Networks (CNNs) [21,22]. While all these methods can perform classification, KNN is limited by metric degeneration in high-dimensional spaces and unstable neighbor selection, which reduces its discriminative power and computational efficiency, ultimately affecting classification accuracy. RF lacks sufficient capacity to model nonlinear coupling within spectral space. MLR, as a linear discriminant model, demonstrates inadequate performance when it lacks sufficient pixels and when the data are complex and nonlinear, particularly in high-dimensional scenarios. CNNs can learn joint spatial–spectral features that require extensive labeled datasets and incur high deployment costs. In comparison, SVM leverages the principle of maximum margin and kernel methods to deliver more stable, interpretable classification results with fewer parameters. By selecting different kernel functions, such as polynomial, radial basis function (RBF), and sigmoid, SVM can construct discriminant boundaries in the implicit feature space, enabling targeted modeling and classification for datasets with varying distribution patterns and degrees of nonlinearity. Consequently, this study employs an SVM with an RBF kernel for hyperspectral image classification.

### 5.1. Datasets

Three benchmark hyperspectral image (HSI) datasets were utilized to comprehensively evaluate the classification performance of SFOAE-SVM. The following section provides a detailed description of these datasets:

1. Salinas: The Salinas dataset was acquired using the Airborne Visible and Infrared Imaging Spectrometer (AVIRIS) sensor in the Salinas Valley, California. The dataset consists of 512 × 217 pixels, covers a spectral range of 0.36 to 2.5 μm, and contains 224 consecutive bands with a spatial resolution of 3.7 m. After excluding 20 absorption bands (108–112, 154–167, 224), 204 bands were used for training. The study area includes 16 surface object categories, such as vegetables, bare land, vineyard fields, and corn weeds. Table 6 presents the quantities and category colors of the training samples and test samples used for classification, and the specified category colors will correspond to those displayed in the classification result images.
2. Trento: The Trento dataset was collected in the rural area south of Trento, Italy, using the AISA Eagle sensor. It comprises 600 × 166 pixels and 63 spectral channels spanning 0.40 to 0.98 μm, with a spatial resolution of 1 m. This dataset includes six categories: apple trees, buildings, ground, forests, vineyards, and roads. Table 7 presents the quantities and category colors of the training samples and test samples used for classification, and the specified category colors will correspond to those displayed in the classification result images.
3. PaviaU: The Pavia University (PaviaU) dataset consists of hyperspectral images acquired from the University of Pavia in northern Italy and its surrounding urban areas. The data were collected using the Reflective Optics System Imaging Spectrometer (ROSIS) sensor, yielding images with a resolution of 610 × 340 pixels. The dataset contains 103 valid spectral bands (from 115 original bands after removing those affected by noise), spanning 0.43 to 0.86 μm, with a spatial resolution of 1.3 m. There are nine land-cover categories: asphalt, grassland, gravel, trees, metal roof, bare soil, asphalt roof, bricks, and shadow. Table 8 presents the quantities and category colors of the training samples and test samples used for classification, and the specified category colors will correspond to those displayed in the classification result images.

### 5.2. Evaluation Metrics

Three main indicators are used to evaluate classification performance: Overall Accuracy (OA) measures the total correct classification, Average Accuracy (AA) calculates the average class recognition rate, and Kappa coefficient quantifies the classification consistency beyond random chance. The visual interpretation of the classification graph provides supplementary qualitative assessment.

### 5.3. Experimental Results

The SFOAE-SVM hyperspectral image classification method proposed in this paper was compared with seven commonly used and traditional hyperspectral image classification methods: K Nearest Neighbors (KNN), Random Forest (RF), Multiple Linear Regression (MLR), Support Vector Machine (SVM), one-dimensional convolutional neural network (1DCNN), two-dimensional convolutional neural network (2DCNN), hybrid three-dimensional and two-dimensional convolutional neural network (HybridSN), particle swarm optimization-based SVM classification method (PSO-SVM), genetic algorithm-based SVM classification method (GA-SVM) and artificial bee colony algorithm-based SVM classification method (ABC-SVM). Using the above ten methods, the three benchmark hyperspectral datasets were classified. For all classification methods, 5% of the labels from three benchmark datasets are used for training, while the remaining 95% are applied for classification. This approach ensures complete isolation between training and test data, enabling a valid verification of the classification performance of different methods. At the same time, the classification results were visualized for intuitive comparison. All classification methods were run independently 10 times, and the average of the obtained results was calculated for comparative analysis. Specifically, the number of neighbors k in KNN is set to 3; the number of decision trees in RF is 50; the regularization coefficient c of MLR is 0.1; SVM adopts the RBF kernel function, and the penalty parameter C and kernel parameter σ are automatically determined via grid search; and for PSO-SVM, GA-SVM, ABC-SVM and SFOAE-SVM, the population size is set to 60 and the maximum number of iterations is set to 100.

#### 5.3.1. The Results of the Salinas Dataset

Table 9 presents a quantitative comparison between SFOAE-SVM and the other ten classification methods. For the 16 land-cover categories in the Salinas dataset, SFOAE-SVM achieved excellent classification accuracy in the vast majority of categories. The algorithm proposed in this paper achieved an OA of 90.29 ± 0.18%, an AA of 93.41 ± 0.14%, and a Kappa of 89.13 ± 0.20%, and had the smallest standard deviation in the indicators for confirming the stability of the algorithm.

**Figure 10:**
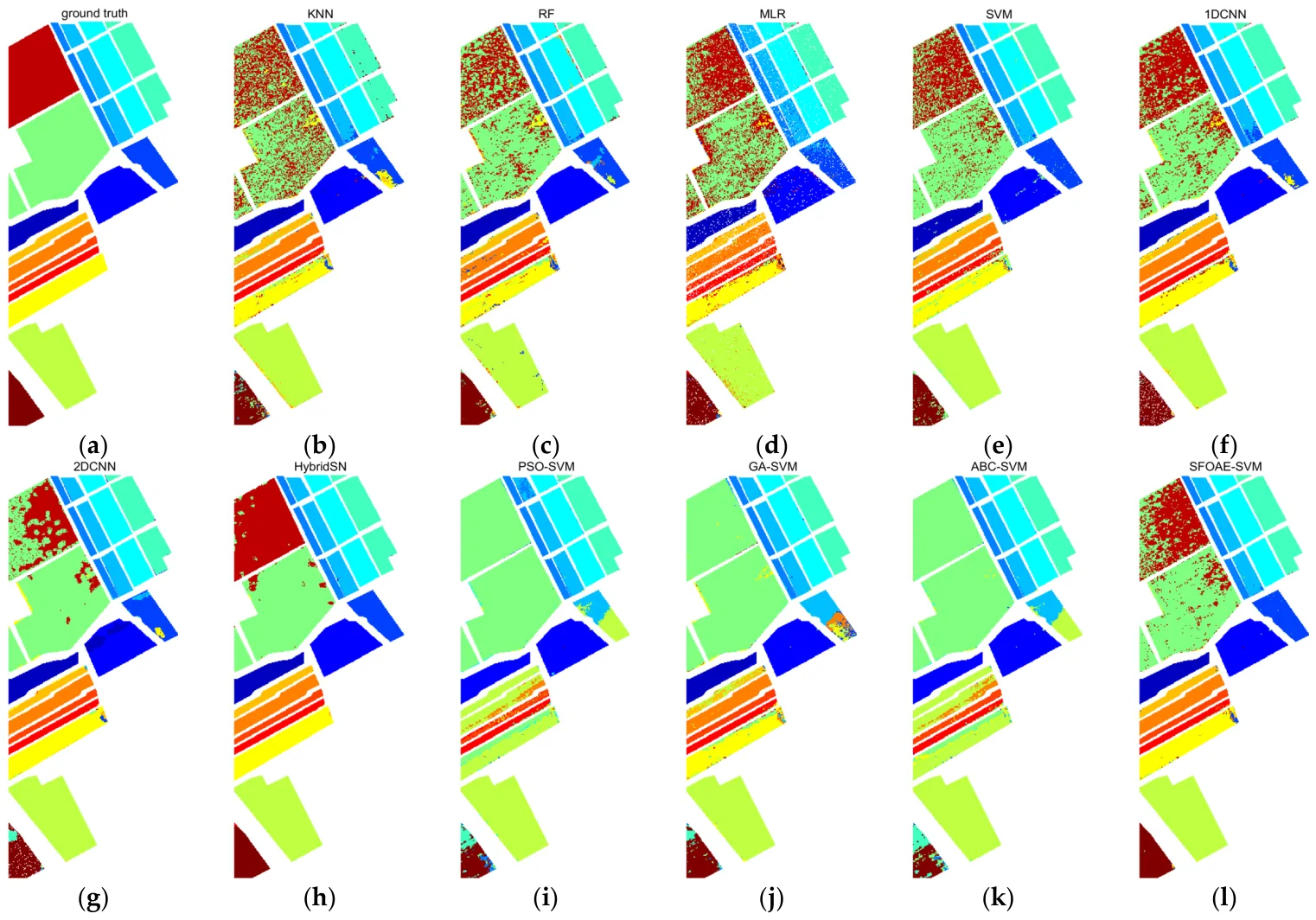
Classification graph of the Salinas dataset: (**a**) Ground truth; (**b**) KNN; (**c**) RF; (**d**) MLR; (**e**) SVM; (**f**) 1DCNN; (**g**) 2DCNN; (**h**) HybridSN; (**i**) PSO-SVM; (**j**) GA-SVM; (**k**) ABC-SVM; (**l**) SFOAE-SVM.

Figure 10 presents a visual comparison using the Salinas dataset, which features agricultural scenes characterized by large fields and multiple parallel crop rows. The spectral similarity among categories and the presence of narrow transitional zones often result in misclassification, leading to local confusion and indistinct boundaries. In the (b) KNN results, numerous noise points are present within the large fields, and the field edges lack smoothness. The (c) RF results demonstrate a reduction in noise within the large field areas; however, some narrow bands are misclassified due to erosion by other categories. The (d) MLR results reveal that the linear discrimination limitations of MLR for high-dimensional, nonlinear hyperspectral image features result in noise points across all land-cover categories, particularly at plot edges, leading to pronounced misclassification. The (e) SVM results, which utilize the RBF kernel to better process high-dimensional nonlinear data, exhibit improved overall performance compared to previous methods. Some narrow strip land plots are classified accurately, and fewer misclassifications occur in large fields, though boundaries between plots with similar spectral characteristics remain unstable. Although the classification performance of (f) 1DCNN is significantly superior to that of traditional machine learning methods, its overall performance is still inferior to 2DCNN and HybridSN due to structural limitations, and misclassification still exists in certain categories. In (g) 2DCNN, spatial information is introduced to assist decision-making, so the classification performance is improved compared with 1DCNN. However, the interference of spatial information also leads to misclassification of some categories. In (h) HybridSN, the spectral–spatial hybrid architecture enables the model to fully extract image information, thus achieving excellent classification performance, and exhibits extremely high accuracy when compared with the ground truth. In contrast, the three optimization algorithm-based methods in (i), (j) and (k) are prone to converge to local optima when processing high-dimensional HSI data and cannot perform efficient global optimization, which results in a large number of misclassifications for easily confused categories except those with obvious features. The (l) SFOAE-SVM results, benefiting from enhanced algorithm optimization and improved penalty and RBF kernel parameters, display reduced noise, minimal overlap and confusion between adjacent strips, and clearer boundaries. Although some noise points persist in the large fields, their frequency is significantly lower than with other methods, and the boundaries are smoother. The improved SFOAE-SVM algorithm more accurately distinguishes objects with similar spectral characteristics while maintaining precise edge depiction, resulting in superior recognition across categories and higher classification accuracy.

#### 5.3.2. The Results of the Trento Dataset

Table 10 presents a quantitative comparison between SFOAE-SVM and the other ten classification methods. For the six land-cover categories of the Trento dataset, SFOAE-SVM achieved excellent classification accuracy in the vast majority of categories. The algorithm proposed in this paper achieved an OA of 92.26 ± 0.20%, an AA of 90.84 ± 0.39%, and a Kappa of 89.67 ± 0.27% and had the smallest standard deviation in the indicators for confirming the stability of the algorithm.

**Figure 11:**
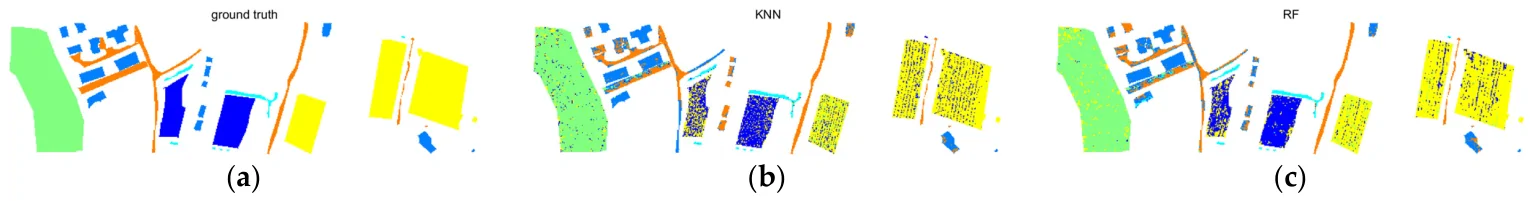

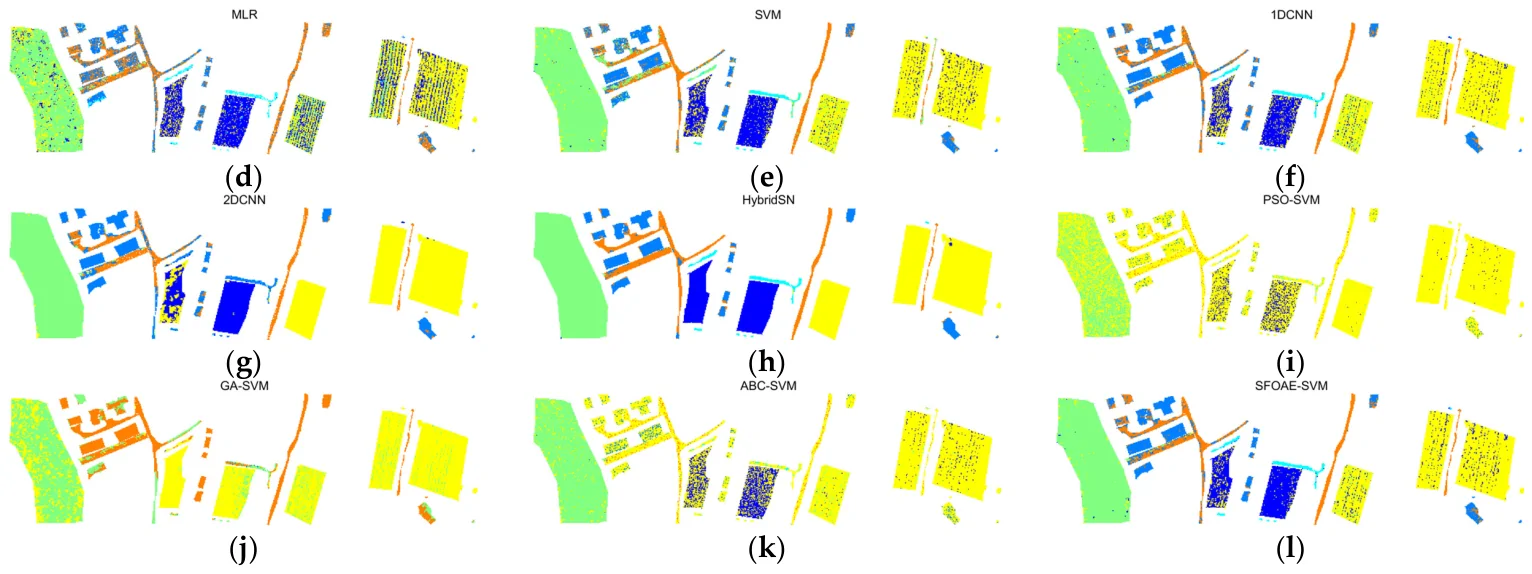
Classification graph of the Trento dataset: (**a**) Ground truth; (**b**) KNN; (**c**) RF; (**d**) MLR; (**e**) SVM; (**f**) 1DCNN; (**g**) 2DCNN; (**h**) HybridSN; (**i**) PSO-SVM; (**j**) GA-SVM; (**k**) ABC-SVM; (**l**) SFOAE-SVM.

Figure 11 presents a visual comparison using the Trento agricultural dataset, which features large fields with multiple parallel crop rows and similar spectral characteristics across categories. The presence of narrow transitional zones increases the likelihood of misclassification, leading to local confusion and indistinct boundaries. In the (b) KNN results, substantial noise is evident in nearly all plots, with severe road discontinuities and significant misclassification, which greatly reduces classification accuracy. The (c) RF results show a marked reduction in noise, although pronounced edge artifacts remain, and the overall accuracy is moderate. The (d) MLR results demonstrate that the linear-discrimination limitations of MLR for high-dimensional, nonlinear hyperspectral features cause adjacent plot strips to erode into each other, increasing intra-plot noise and misclassification and reducing classification accuracy. The (e) SVM results, using the RBF kernel for high-dimensional nonlinear data, further reduce noise and inter-plot erosion, yielding clearer plot boundaries than previous methods. In the classification results of (f) 1DCNN, the classification performance shows a significant advantage over traditional machine learning methods. However, constrained by its network architecture, the overall performance is inferior to that of 2DCNN and HybridSN, and misclassification still occurs for certain categories. In (g) 2DCNN, since spatial information is partially incorporated as a reference for classification, the classification performance is improved to a certain extent compared with 1DCNN. Nevertheless, the introduction of spatial information may interfere with the model’s decision-making, leading to high misclassification probability for certain categories. In (h) HybridSN, the proposed spectral–spatial hybrid network architecture can fully exploit the information of images to obtain superior classification results, and it can be observed that this method achieves extremely high classification accuracy compared with the ground truth. In (i), (j) and (k), the three classification methods based on optimization algorithms are all prone to fall into local optima when processing high-dimensional data such as HSI and cannot effectively search for near-optimal parameters. Except for categories with distinct features, numerous misclassifications occur among other easily confused categories. The (l) SFOAE-SVM results benefit from improved algorithm optimization, yielding superior penalty and RBF kernel parameters, which effectively eliminate classification noise. The resulting plots are complete and coherent, with smooth, well-defined edges and only a few breakpoints in narrow strips. The spatial layout closely matches the ground truth. The improved SFOAE-SVM algorithm demonstrates superior ability to distinguish objects with similar spectral characteristics while maintaining precise edge delineation. It excels in block consistency and preservation of fine linear structures, resulting in higher classification accuracy.

#### 5.3.3. The Results of the PaviaU Dataset

Table 11 provides a quantitative comparison of SFOAE-SVM with ten other classification methods. For the nine land feature types in the PaviaU dataset, SFOAE-SVM achieved high classification accuracy in most categories. While it did not attain the highest accuracy in every category, the recognition accuracy distribution remained concentrated across all categories, with no significantly underperforming classes. The model exhibited balanced performance across different classes. The proposed algorithm achieved an overall accuracy (OA) of 91.98 ± 0.49%, an average accuracy (AA) of 89.11 ± 0.84%, and a Kappa coefficient of 89.27 ± 0.70%. Additionally, it demonstrated the smallest standard deviation among the evaluated indicators, confirming the algorithm’s stability.

**Figure 12:**
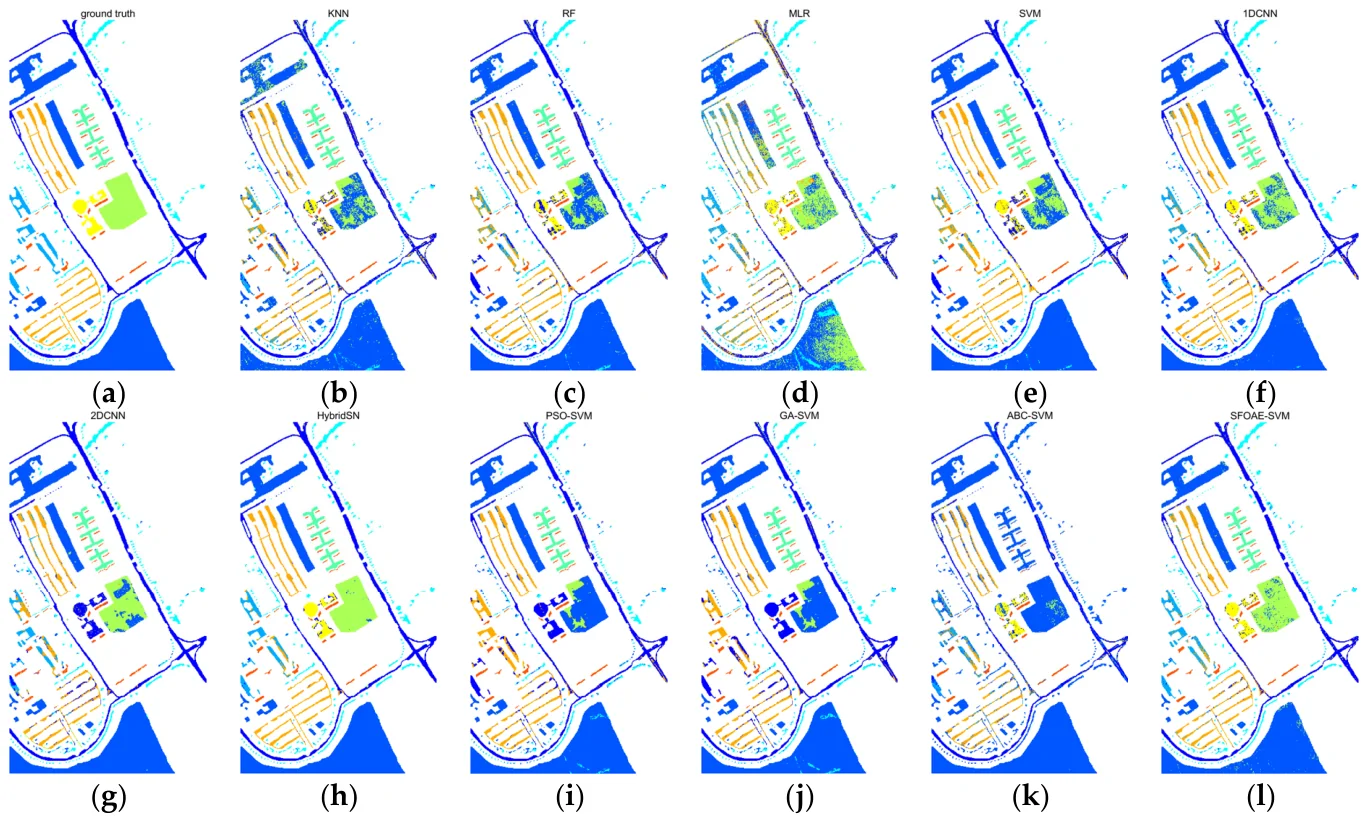
Classification graph of the PaviaU dataset: (**a**) Ground truth; (**b**) KNN; (**c**) RF; (**d**) MLR; (**e**) SVM; (**f**) 1DCNN; (**g**) 2DCNN; (**h**) HybridSN; (**i**) PSO-SVM; (**j**) GA-SVM; (**k**) ABC-SVM; (**l**) SFOAE-SVM.

Figure 12 presents a visual comparison of classification results on the PaviaU dataset, which comprises urban scenes characterized by similar materials and spectral patterns across categories, as well as narrow plots. These factors often contribute to misclassification, resulting in local confusion and indistinct boundaries. The visual classification results indicate that both (b) KNN and (c) RF exhibit significant noise and extensive regions of incorrect classification. Additionally, breakpoints in the narrow plots are prominent, and edge delineation is poor. KNN, in particular, is more vulnerable to local anomalous samples, which further reduces overall classification accuracy. In (d) MLR, the inherent limitations of linear discrimination in MLR for high-dimensional and nonlinear features of hyperspectral images result in numerous misclassifications among objects with similar spectral characteristics. This also leads to increased noise and blurred edges in the narrow plot category. The (e) SVM results, which utilize the RBF kernel to enhance processing of high-dimensional nonlinear data, show improved overall performance compared to previous methods. In the classification results of (f) 1DCNN, benefiting from the advantages of convolutional neural networks, most categories can be correctly classified, showing a significant advantage over traditional machine learning methods. However, constrained by its network architecture, the overall performance of 1DCNN is weaker than that of 2DCNN and HybridSN. For (g) 2DCNN, since spatial information is partially introduced as a reference for classification, the classification performance is improved to a certain extent compared with 1DCNN, but misclassification still occurs. In (h) HybridSN, the spectral–spatial hybrid network architecture can fully exploit image information to obtain better classification results, and it can be observed that extremely high classification accuracy is achieved compared with the ground truth. The three classification methods based on optimization algorithms in (i), (j), and (k) are all prone to fall into local optima when processing high-dimensional data such as HSI and cannot effectively search for near-optimal parameters. Except for categories with obvious features, more misclassifications occur for other easily confused categories. The (l) SFOAE-SVM results, achieved through enhanced algorithm optimization and improved penalty and RBF kernel parameters, display reduced noise, minimal large-scale misclassification, limited misclassification spread, and the absence of obvious breakpoints in narrow plot categories, with smoother edges. In summary, the improved SFOAE-SVM algorithm more accurately distinguishes objects with similar spectral characteristics, maintains precise edge depiction, preserves the integrity of the linear structure, and demonstrates excellent performance in category identification, thereby achieving higher classification accuracy.

### 5.4. Quantitative Analysis

To objectively evaluate the performance advantage of SFOAE-SVM over other comparison algorithms at the statistical level, this study conducted a significance level test (p<0.05) on the overall accuracy (OA), average accuracy (AA) and Kappa coefficient obtained from 10 independent runs. The calculated *p*-values are shown in Table 12, which presents the test results on three datasets and the number of cases that satisfy p<0.05. Compared with conventional machine learning methods of the same type, the *p*-values of SFOAE-SVM against KNN, RF, MLR and unoptimized standard SVM on all three datasets are all less than 0.05, indicating that in the pure spectral dimension, the proposed algorithm has a significant statistical advantage over traditional statistical classifiers and untuned kernel methods. Compared with PSO-SVM, GA-SVM and ABC-SVM, which are SVM optimized by intelligent optimization algorithms, the *p*-values of SFOAE-SVM against these methods all reach the significance level of p<0.05, which verifies that SFOAE-SVM performs excellently in global optimization and in avoiding local optima and can explore better parameter combinations, thus achieving superior classification performance. When compared with deep learning methods, the proposed algorithm still maintains an advantage in the comparison against 1DCNN; however, it does not obtain a significant advantage against 2DCNN and shows a slight performance gap in some datasets. This result indicates that classification relying solely on spectral data has inherent limitations compared with 2DCNN, which can exploit the two-dimensional spatial information of adjacent pixels, thus leading to a certain gap in the final classification performance. There is also an objective gap when compared with HybridSN, a spectral–spatial deep learning model that can utilize complete spatial and spectral information, and the statistical results show a considerable performance gap. This result is consistent with the physical principle of hyperspectral classification: the difference in information dimension makes it difficult for SFOAE-SVM, which only uses one-dimensional spectral data, to surpass the hybrid 3D network in terms of the upper limit of absolute accuracy.

Table 13 presents the running time of each compared algorithm on three datasets, which reflects the trade-off between classification accuracy and computational cost of the algorithms. Traditional machine learning methods, as well as one-dimensional and two-dimensional neural networks, do not require complex global optimization iterations; thus, their single-run time consumption is relatively low. However, these low time-cost methods show certain deficiencies in classification accuracy. The running time of SFOAE-SVM on the three datasets is significantly increased compared with the comparison methods. Compared with unoptimized SVM, SFOAE-SVM adds the time overhead of global hyperparameter optimization; compared with other similar optimization-based SVMs, the computational time consumption of SFOAE-SVM increases due to the integration of a multi-strategy collaborative iteration mechanism. Nevertheless, this moderate increase in time cost enables the algorithm to achieve a significant improvement in classification accuracy, as verified in Table 12. As a representative of spectral–spatial joint networks, HybridSN achieves higher classification accuracy, but its computational overhead is excessively high. From the perspective of practical application, HybridSN fails to achieve an effective balance between running efficiency and classification accuracy; although its classification accuracy is considerably high, its excessively long running time and high computational cost limit its practical application scenarios. Based on the comprehensive analysis of running time and classification accuracy, SFOAE-SVM, with a lightweight architecture that does not rely on complex spatial information and complex 3D convolution calculation, successfully balances optimization time consumption and classification accuracy. In single-spectrum application scenarios, SFOAE-SVM achieves better accuracy than traditional networks and demonstrates more cost-effective computational efficiency and satisfactory classification performance compared with 3D convolution networks.

### 5.5. Overall Analysis

Although the proposed SFOAE-SVM in this paper achieves the globally optimal overall classification performance in terms of Overall Accuracy (OA), Average Accuracy (AA), and Kappa coefficient, with the exception of HybridSN, several comparative algorithms attain relatively higher per-class classification accuracy on a small number of specific land-cover classes. This phenomenon reflects the influence of the inherent spectral similarity characteristics of hyperspectral data on classification results.

At the algorithm design level, this paper adopts the overall classification performance as the fitness function to guide hyperparameter search. The core objective of this strategy is to enable the classifier to construct a nonlinear decision boundary that balances the discriminative capability for all land-cover classes across the entire feature space. However, in real-world hyperspectral images, the problems of serious intra-class spectral variation, inter-class spectral confusion, and class imbalance commonly exist among different land-cover types. Under the interference of these objective factors, the decision boundaries of some comparative algorithms are prone to skew toward specific spectral distributions. When a certain land-cover class has high local sample density or prominent spectral features, the algorithm tends to expand the decision space for this class and preferentially assigns ambiguous pixels and mixed pixels in the junction area to this category. Although this local skewing of the decision boundary happens to improve the per-class classification accuracy of this specific class objectively, it essentially comes at the cost of sacrificing the discriminative accuracy of adjacent overlapping classes and ultimately leads to a decrease in overall classification performance.

In contrast, the SFOAE-SVM proposed in this paper effectively suppresses decision boundary deviation caused by sample imbalance and mixed pixels via a multi-strategy collaborative optimization mechanism. Instead of sacrificing global discriminative power to pursue the extreme maximum accuracy of a single class, the proposed method achieves balanced classification performance for all classes under the constraints of complex spectral overlap and spatial heterogeneity.

## 6. Conclusions

A novel intelligent optimization algorithm, SFOAE-SVM, is introduced, integrating a support vector machine (SVM) for image classification. The starfish optimization algorithm is enhanced through four key steps that significantly accelerate convergence, optimize outputs, and improve robustness. The SVM classifier is combined with the enhanced starfish optimization algorithm (SFOAE), and the intelligent optimization algorithm is utilized to identify the optimal parameter combination. This approach effectively addresses the low classification efficiency and poor accuracy often observed in traditional SVMs. Experimental results indicate that the improved SFOAE-SVM algorithm outperforms commonly used classical algorithms and is specifically designed for optimizing SVM classification in hyperspectral images. The findings demonstrate that the proposed method achieves superior classification performance, enables accurate classification, and enhances algorithmic stability.

Although the SFOAE-SVM algorithm proposed in this paper achieves improved classification performance across multiple hyperspectral datasets, comprehensive comparisons on three benchmark datasets indicate that SFOAE-SVM delivers relatively superior performance, specifically on the low-dimensional Trento dataset. When applied to higher-dimensional datasets, the algorithm still encounters considerable challenges, which slightly undermine its optimization capability and classification performance. In addition, given the black-box optimization strategy adopted in this work, obtaining the objective function value via five-fold cross-validation will increase the total optimization time. Nevertheless, this approach still effectively reduces the workload and time consumption associated with manual hyperparameter tuning.

Future research will focus on fusing spectral and spectral feature image features to minimize redundant data, followed by classification of hyperspectral images and optimization of the objective function to further enhance the SFOAE-SVM algorithm’s classification capability.

## Figures and Tables

**Table 1:** Comparison Results Between SFOA and SFOAE.

Module	SFOA	SFOAE	Structural Difference
Initialization Phase	Random Distribution	Uniform distribution of PWLCM (Equation (24))	Random → Uniform
Exploration Phase	Stochastic Exploration	Adaptive adjustment of ASDW along with iterations (Equations (25) and (30))	Random → Adaptive
Mutation Strategy	None	Sudden position mutation of QTE (Equation (33))	Fixed → Prone to escaping local optima
Population Information Interaction Strategy	None	Population information interaction via Bi-JADE (Equation (39))	No interaction → Information interaction increases population diversity

**Table 2:** CEC2022 benchmark functions.

	No.	Functions	$F_{i}^{*}$	D	Search Range
Unimodal Function	1	Shifted and full Rotated Zakharov Function	300	20	${\left[-100,100\right]}^{D}$
Basic Functions	2	Shifted and full Rotated Rosenbrock’s Function	400	20	${\left[-100,100\right]}^{D}$
	3	Shifted and full Rotated Expanded Schaffer’s f6 Function	600	20	${\left[-100,100\right]}^{D}$
	4	Shifted and full Rotated Non-Continuous Rastrigin’s Function	800	20	${\left[-100,100\right]}^{D}$
	5	Shifted and full Rotated Levy Function	900	20	${\left[-100,100\right]}^{D}$
Hybrid Functions	6	Hybrid Function 1 $\left(N=3\right)$	1800	20	${\left[-100,100\right]}^{D}$
	7	Hybrid Function 2 $\left(N=6\right)$	2000	20	${\left[-100,100\right]}^{D}$
	8	Hybrid Function 3 $\left(N=5\right)$	2200	20	${\left[-100,100\right]}^{D}$
Composition Functions	9	Composition Function 1 $\left(N=5\right)$	2300	20	${\left[-100,100\right]}^{D}$
	10	Composition Function 2 $\left(N=4\right)$	2400	20	${\left[-100,100\right]}^{D}$
	11	Composition Function 3 $\left(N=5\right)$	2600	20	${\left[-100,100\right]}^{D}$
	12	Composition Function 4 $\left(N=6\right)$	2700	20	${\left[-100,100\right]}^{D}$

**Table 3:** Statistical results on the CEC2022 benchmark functions.

f	Results	SFOAE	SFOA	GOOSE	HHO	WOA	GJO	SCSO	OOA	SABO
F1	min	300	660.9676	1153.5398	4714.6965	11,115.2649	2895.5633	2000.2511	21,570.7091	19,634.9684
	mean	300	1405.7695	11,983.4035	12,444.8816	25,688.6039	13,609.0574	11,516.6917	38,827.2480	28,435.7868
	worse	300	2635.2881	40,490.2990	36,680.3396	54,479.8387	22,759.8994	21,452.8439	72,290.3670	39,340.4110
F2	min	400	416.8024	449.1014	472.3952	457.0800	491.8534	436.3255	1279.3465	544.2772
	mean	434.2142	458.5439	493.2930	512.2064	542.0654	578.7847	527.0893	2112.2787	665.4226
	worse	449.0845	499.8324	610.7679	605.9501	685.5719	750.6033	624.5535	4034.6071	941.9349
F3	min	600	608.8977	647.1946	633.6562	646.9638	608.0804	622.7539	652.2530	621.2546
	mean	600	622.3643	670.3600	656.3948	665.6309	623.1062	646.0557	671.6147	635.7757
	worse	600.6564	634.8959	690.6728	671.7635	697.4584	639.9058	671.8366	688.8478	654.9790
F4	min	812.9345	897.0652	884.5716	865.0741	864.3644	825.6159	859.4986	912.8699	913.1591
	mean	822.9020	943.7868	931.1398	883.8433	927.3292	895.9864	894.5250	951.2777	943.8225
	worse	886.9335	968.6901	1002.9707	929.1833	998.4080	968.2585	930.1172	992.0791	981.5393
F5	min	900	1179.0951	2452.4385	2194.3652	1553.9790	1167.1040	1269.6733	2357.3297	1033.2657
	mean	900.0452	2010.2123	3748.4673	2811.2173	3706.9720	1700.9651	2184.6073	3063.8308	1730.8629
	worse	900.5439	3183.0851	5995.9244	3509.1310	6894.5855	2720.4330	2773.1098	3898.4517	2785.9276
F6	min	1807.2910	3638.0235	1991.1526	22,278.7373	55,683.9174	18,369.2551	2507.2900	128,535,667.1000	344,604.0166
	mean	1858.4319	20,407.7640	6927.9578	129,752.9897	488,107.9597	12,037,163.0600	1,945,526.5840	1,188,223,907	9,973,754.1160
	worse	1931.1686	79,864.0139	20,462.2226	285,917.6313	2,278,409.5980	58,648,165.2700	40,939,584.2200	3,612,743,614	53,608,938.5000
F7	min	2000	2080.2856	2107.5732	2087.1668	2076.1521	2052.0245	2065.6464	2117.5503	2119.3450
	mean	2016.0530	2113.1403	2270.5585	2167.9246	2203.7592	2111.3852	2132.2952	2176.8187	2210.3865
	worse	2050.0336	2184.9653	2577.8504	2324.4883	2391.5014	2185.8575	2219.8293	2272.3688	2318.9180
F8	min	2220.1638	2234.3579	2232.9163	2232.4925	2228.9766	2226.4330	2228.8316	2233.5988	2247.2557
	mean	2240.5088	2244.0325	2541.6428	2259.0597	2287.5993	2259.1601	2298.0547	2274.4026	2363.4796
	worse	2362.9540	2253.0923	2963.5094	2464.2305	2382.6196	2368.8916	2472.7181	2531.7746	2577.1607
F9	min	2480.7813	2480.7972	2486.8829	2483.9279	2496.1564	2507.2681	2493.6204	2860.4447	2590.3079
	mean	2480.7813	2480.9099	2572.6620	2513.1680	2562.8864	2576.8401	2537.2649	3184.3381	2690.1655
	worse	2480.7813	2481.6823	2681.6038	2597.2875	2693.6048	2682.9723	2644.2775	3545.0267	2792.0042
F10	min	2400.1561	2500.7917	2500.6545	2500.8825	2500.9273	2501.2578	2500.7194	2592.1319	2990.3472
	mean	2512.1793	3912.0172	4704.4070	3946.9666	4164.2716	3587.6575	3257.0705	5410.9128	6387.7257
	worse	2763.0389	6338.1254	5963.7606	5514.4541	6433.0821	6665.3030	5527.2017	7060.0977	7418.1879
F11	min	2900	3394.4824	2900.5866	2682.9545	3118.1518	4204.4881	3265.5909	7182.5216	3506.1954
	mean	2900	3586.1902	55,399.6612	3188.1770	3500.6034	5415.3335	4215.6139	8714.9498	4985.5043
	worse	2900	3984.4606	181,847.7691	3785.9388	6379.4158	7550.2968	6289.4147	9862.1223	7376.0812
F12	min	2933.5741	2936.6517	3155.5676	2990.3427	2959.4937	2948.6982	2963.4957	3496.3537	2981.7620
	mean	2952.7562	2948.1142	3543.0356	3104.2937	3071.7941	2997.8738	3015.6510	3737.0722	3064.1012
	worse	2998.5765	3015.6547	4093.9485	3303.8651	3320.2041	3093.2951	3123.0516	4113.1550	3148.6886

**Table 4:** Results of the Wilcoxon rank-sum test on CEC2022.

f	SFOA	GOOSE	HHO	WOA	GJO	SCSO	OOA	SABO
F1	5.14 × 10^−12^	5.14 × 10^−12^	5.14 × 10^−12^	5.14 × 10^−12^	5.14 × 10^−12^	5.14 × 10^−12^	5.14 × 10^−12^	5.14 × 10^−12^
F2	1.42 × 10^−5^	1.44 × 10^−10^	2.17 × 10^−11^	2.17 × 10^−11^	2.17 × 10^−11^	1.44 × 10^−10^	2.17 × 10^−11^	2.17 × 10^−11^
F3	1.27 × 10^−11^	1.27 × 10^−11^	1.27 × 10^−11^	1.27 × 10^−11^	1.27 × 10^−11^	1.27 × 10^−11^	1.27 × 10^−11^	1.27 × 10^−11^
F4	3.01 × 10^−11^	3.01 × 10^−11^	4.06 × 10^−11^	3.01 × 10^−11^	5.47 × 10^−11^	4.06 × 10^−11^	3.01 × 10^−11^	3.01 × 10^−11^
F5	1.53 × 10^−11^	1.53 × 10^−11^	1.53 × 10^−11^	1.53 × 10^−11^	1.53 × 10^−11^	1.53 × 10^−11^	1.53 × 10^−11^	1.53 × 10^−11^
F6	3.02 × 10^−11^	3.02 × 10^−11^	3.02 × 10^−11^	3.02 × 10^−11^	3.02 × 10^−11^	3.02 × 10^−11^	3.02 × 10^−11^	3.02 × 10^−11^
F7	3.02 × 10^−11^	3.02 × 10^−11^	3.02 × 10^−11^	3.02 × 10^−11^	3.02 × 10^−11^	3.02 × 10^−11^	3.02 × 10^−11^	3.02 × 10^−11^
F8	1.60 × 10^−7^	2.15 × 10^−10^	1.87 × 10^−7^	4.69 × 10^−8^	6.53 × 10^−7^	1.73 × 10^−7^	7.69 × 10^−8^	1.56 × 10^−8^
F9	1.21 × 10^−12^	1.21 × 10^−12^	1.21 × 10^−12^	1.21 × 10^−12^	1.21 × 10^−12^	1.21 × 10^−12^	1.21 × 10^−12^	1.21 × 10^−12^
F10	1.87 × 10^−5^	2.92 × 10^−9^	1.69 × 10^−9^	5.97 × 10^−9^	2.60 × 10^−8^	1.86 × 10^−6^	3.69 × 10^−11^	3.02 × 10^−11^
F11	4.08 × 10^−12^	4.08 × 10^−12^	4.08 × 10^−12^	4.08 × 10^−12^	4.08 × 10^−12^	4.08 × 10^−12^	4.08 × 10^−12^	4.08 × 10^−12^
F12	9.71 × 10^−1^	3.02 × 10^−11^	5.49 × 10^−11^	1.09 × 10^−10^	1.17 × 10^−9^	7.39 × 10^−11^	3.02 × 10^−11^	3.02 × 10^−11^
	11	12	12	12	12	12	12	12

**Table 5:** Ablation results under different improvement strategies.

f	Results	SFOAE	SFOAE_BiJADE	SFOAE_QTE	SFOAE_ASDW	SFOAE_PWLCM	SFOA
F1	min	300	300	300.1284	563.9180	592.5057	578.9468
F1	mean	300	300	300.3102	1660.9812	1563.4326	1547.1476
F1	worse	300	300	300.4687	4072.5626	4972.9841	4184.0566
F2	min	400	400	404.3234	438.7505	413.8931	431.4297
F2	mean	434.0746	440.2056	441.6431	458.3711	457.1928	456.5244
F2	worse	449.0845	449.0845	472.0081	511.8824	487.4251	511.0970
F5	min	900	900	900.0503	1331.1696	1097.5028	1092.9702
F5	mean	900.0755	900.1058	1249.8679	1882.1827	1822.3200	2096.3349
F5	worse	900.5439	900.9086	3371.6209	3716.0220	4110.6849	3478.2082
F6	min	1806.1889	1804.4242	1980.6007	2316.4445	5362.4376	3606.0871
F6	mean	1869.3648	1848.9593	3885.9424	17,087.9825	23,246.2841	18,746.7034
F6	worse	1958.3567	1901.5823	25,224.1401	37,508.9737	35,564.9505	43,797.6613
F7	min	2000.3122	2000.0000	2021.0335	2078.6869	2068.9648	2077.3315
F7	mean	2017.2884	2007.5194	2062.4267	2115.4161	2114.5499	2116.8312
F7	worse	2140.7026	2021.1720	2166.9247	2160.1869	2198.5787	2164.6731
F9	min	2480.7813	2480.7813	2480.7822	2480.8290	2480.7879	2480.7908
F9	mean	2480.7813	2480.7813	2480.7846	2481.0010	2480.8839	2480.8825
F9	worse	2480.7813	2480.7813	2480.7920	2481.2930	2481.0489	2481.0822

**Table 6:** Description of categories in the Salinas dataset and the number of training and testing samples for each category.

No.	Color	Class	Train	Test
1		Brocoli_green_weeds_1	201	1808
2		Brocoli_green_weeds_2	373	3353
3		Fallow	198	1778
4		Fallow_rough_plow	139	1255
5		Fallow_smooth	268	2410
6		Stubble	396	3563
7		Celery	358	3221
8		Grapes_untrained	1127	10,144
9		Soil_vinyard_develop	620	5583
10		Corn_senesced_green_weeds	328	2950
11		Lettuce_romaine_4wk	107	961
12		Lettuce_romaine_5wk	193	1734
13		Lettuce_romaine_6wk	92	824
14		Lettuce_romaine_7wk	107	963
15		Vinyard_untrained	727	6541
16		Vinyard_vertical_trellis	181	1626
Total			5413	48,716

**Table 7:** Description of categories in the Trento dataset and the number of training and testing samples for each category.

No.	Color	Class	Train	Test
1		Apple trees	403	3631
2		Buildings	290	2613
3		Ground	48	431
4		Wood	912	8211
5		Vineyard	1050	9451
6		Roads	337	3037
Total			3041	27,373

**Table 8:** Description of categories in the PaviaU dataset and the number of training and testing samples for each category.

No.	Color	Class	Train	Test
1		Asphalt	663	5968
2		Meadows	1865	16,784
3		Gravel	210	1889
4		Trees	306	2758
5		Painted metal sheets	135	1211
6		Bare Soil	503	4526
7		Bitumen	133	1197
8		Self-Blocking Bricks	368	3314
9		Shadows	95	852
Total			4278	38,498

**Table 9:** The comparison results of the Salinas dataset.

Class	KNN	RF	MLR	SVM	1DCNN	2DCNN	HybridSN	PSO_SVM	GA_SVM	ABC_SVM	SFOAE_SVM
1	95.89 ± 1.26	97.66 ± 0.93	97.75 ± 0.50	97.56 ± 0.19	97.25 ± 1.24	93.68 ± 8.91	100.00 ± 0.00	7.66 ± 24.21	97.25 ± 0.89	0.00 ± 0.00	98.93 ± 0.26
2	98.54 ± 1.07	99.84 ± 0.07	95.97 ± 1.19	96.85 ± 0.42	98.59 ± 3.30	99.46 ± 0.89	99.98 ± 0.05	97.29 ± 6.95	99.87 ± 0.08	99.76 ± 0.03	98.01 ± 0.32
3	89.10 ± 4.84	89.17 ± 1.30	89.34 ± 2.36	96.20 ± 0.74	79.41 ± 9.74	76.60 ± 18.79	99.99 ± 0.03	5.94 ± 18.79	25.20 ± 26.42	0.00 ± 0.00	98.82 ± 0.29
4	99.01 ± 0.52	94.30 ± 1.00	97.08 ± 1.03	94.89 ± 1.14	98.32 ± 0.70	98.35 ± 1.83	98.94 ± 2.45	96.38 ± 11.00	97.32 ± 0.87	99.71 ± 0.00	97.92 ± 0.72
5	95.35 ± 1.39	95.39 ± 0.73	91.47 ± 1.62	95.86 ± 0.47	97.11 ± 1.01	95.90 ± 2.24	98.77 ± 0.79	91.50 ± 2.07	98.92 ± 0.37	98.27 ± 0.03	96.74 ± 0.45
6	99.39 ± 0.19	97.88 ± 0.52	99.24 ± 0.16	95.70 ± 0.35	99.54 ± 0.21	99.95 ± 0.14	99.98 ± 0.05	96.35 ± 10.91	99.76 ± 0.02	99.85 ± 0.01	98.09 ± 0.22
7	98.73 ± 0.43	98.89 ± 0.35	99.78 ± 0.05	98.17 ± 0.29	99.23 ± 0.21	99.71 ± 0.35	99.92 ± 0.24	98.55 ± 2.61	99.35 ± 0.15	99.29 ± 0.07	99.07 ± 0.17
8	72.41 ± 3.37	82.99 ± 1.41	68.21 ± 2.48	86.38 ± 0.70	81.66 ± 8.97	81.25 ± 14.11	96.36 ± 2.18	98.10 ± 1.84	96.77 ± 2.82	98.50 ± 0.02	88.45 ± 0.74
9	96.82 ± 0.61	98.51 ± 0.15	98.82 ± 0.29	97.80 ± 0.24	98.63 ± 0.48	98.92 ± 1.54	100.00 ± 0.00	99.29 ± 1.79	99.37 ± 0.05	99.95 ± 0.01	98.61 ± 0.25
10	81.64 ± 2.30	79.63 ± 1.12	86.20 ± 1.57	82.61 ± 1.09	83.96 ± 3.02	92.44 ± 2.63	99.61 ± 0.43	7.65 ± 13.73	82.77 ± 3.95	6.37 ± 0.43	88.18 ± 0.76
11	80.81 ± 5.37	86.19 ± 2.05	95.12 ± 1.11	87.31 ± 2.14	82.69 ± 9.89	63.13 ± 35.03	99.79 ± 0.30	4.63 ± 14.63	61.38 ± 14.05	0.00 ± 0.00	93.26 ± 0.97
12	98.31 ± 1.01	96.05 ± 0.65	93.84 ± 0.78	97.21 ± 0.46	99.50 ± 0.61	98.53 ± 2.37	99.99 ± 0.02	25.20 ± 13.98	99.54 ± 0.20	30.52 ± 2.50	99.01 ± 0.22
13	97.51 ± 0.84	97.01 ± 2.18	91.62 ± 2.55	93.72 ± 0.92	97.94 ± 0.71	99.74 ± 0.43	99.97 ± 0.11	69.15 ± 6.29	95.51 ± 1.21	91.40 ± 2.88	96.94 ± 0.57
14	89.79 ± 1.62	91.91 ± 1.69	88.65 ± 1.69	80.73 ± 1.72	90.70 ± 2.83	98.31 ± 1.18	99.66 ± 0.53	86.03 ± 16.37	92.86 ± 0.67	88.93 ± 1.03	87.21 ± 1.30
15	54.12 ± 3.61	53.50 ± 1.84	66.95 ± 2.68	63.67 ± 0.96	56.68 ± 12.95	76.30 ± 18.13	98.07 ± 1.43	4.27 ± 13.52	6.70 ± 21.20	0.00 ± 0.00	62.02 ± 1.28
16	77.79 ± 5.43	91.27 ± 1.45	95.28 ± 0.72	84.67 ± 1.70	96.23 ± 1.54	94.53 ± 2.76	99.45 ± 0.46	68.38 ± 14.09	88.93 ± 3.24	52.77 ± 2.78	93.35 ± 1.13
OA	84.16 ± 0.80	86.80 ± 0.21	85.66 ± 0.44	88.42 ± 0.37	87.31 ± 0.76	89.94 ± 1.71	98.83 ± 0.37	66.75 ± 1.74	81.29 ± 3.86	66.67 ± 0.14	90.29 ± 0.18
AA	89.08 ± 0.73	90.64 ± 0.19	90.96 ± 0.30	90.58 ± 0.50	91.09 ± 1.02	91.68 ± 2.77	99.41 ± 0.18	59.77 ± 1.66	83.84 ± 4.18	60.33 ± 0.24	93.41 ± 0.14
Kappa	82.34 ± 0.89	85.26 ± 0.24	84.04 ± 0.48	87.00 ± 0.42	85.84 ± 0.84	88.80 ± 1.90	98.70 ± 0.41	62.02 ± 1.83	78.91 ± 4.38	61.95 ± 0.17	89.13 ± 0.20

**Table 10:** The comparison results of the Trento dataset.

Class	KNN	RF	MLR	SVM	1DCNN	2DCNN	HybridSN	PSO_SVM	GA_SVM	ABC_SVM	SFOAE_SVM
1	73.73 ± 7.08	78.12 ± 1.66	76.16 ± 2.05	80.14 ± 1.58	75.30 ± 10.32	77.66 ± 11.54	99.69 ± 0.26	25.39 ± 2.49	0.00 ± 0.00	53.30 ± 1.80	87.16 ± 0.83
2	76.08 ± 5.25	76.41 ± 1.85	69.37 ± 4.72	77.73 ± 2.04	85.53 ± 4.30	80.80 ± 8.20	93.89 ± 1.19	10.18 ± 0.11	8.61 ± 7.49	18.34 ± 1.37	86.90 ± 1.50
3	87.70 ± 3.59	94.24 ± 3.01	94.41 ± 0.76	64.91 ± 3.15	89.26 ± 2.92	7.61 ± 20.70	95.57 ± 2.26	10.79 ± 0.85	2.78 ± 1.61	13.17 ± 1.83	96.30 ± 0.82
4	93.46 ± 1.07	92.85 ± 0.47	76.37 ± 3.70	96.70 ± 0.28	95.57 ± 1.35	99.36 ± 0.39	99.90 ± 0.07	56.61 ± 2.80	68.63 ± 1.14	89.58 ± 1.54	97.91 ± 0.22
5	78.83 ± 3.13	89.09 ± 1.05	65.57 ± 2.42	89.72 ± 0.84	88.29 ± 5.14	99.88 ± 0.08	99.87 ± 0.09	98.64 ± 0.32	92.49 ± 0.42	94.09 ± 0.55	93.25 ± 0.49
6	71.80 ± 4.82	73.95 ± 2.42	68.75 ± 3.50	68.38 ± 1.89	77.46 ± 3.64	77.31 ± 5.62	91.69 ± 1.92	10.00 ± 0.02	84.25 ± 0.36	12.95 ± 0.58	83.52 ± 1.84
OA	81.70 ± 0.52	86.03 ± 0.43	71.33 ± 1.45	86.76 ± 0.29	87.36 ± 1.93	91.09 ± 1.63	98.35 ± 0.20	56.97 ± 1.05	61.77 ± 0.23	70.20 ± 0.71	92.26 ± 0.20
AA	80.27 ± 1.04	84.11 ± 0.63	75.11 ± 0.96	79.60 ± 0.86	85.23 ± 1.97	73.77 ± 4.45	96.77 ± 0.56	35.27 ± 0.80	41.37 ± 0.31	46.90 ± 0.80	90.84 ± 0.39
Kappa	75.78 ± 0.74	81.34 ± 0.56	62.20 ± 1.84	82.14 ± 0.42	83.12 ± 2.57	87.97 ± 2.25	97.80 ± 0.27	36.13 ± 1.68	45.95 ± 0.37	56.92 ± 1.09	89.67 ± 0.27

**Table 11:** The comparison results of the PaviaU dataset.

Class	KNN	RF	MLR	SVM	1DCNN	2DCNN	HybridSN	PSO_SVM	GA_SVM	ABC_SVM	SFOAE_SVM
1	80.34 ± 3.48	88.67 ± 0.76	59.99 ± 2.50	90.63 ± 1.12	92.28 ± 1.30	97.81 ± 1.59	98.60 ± 0.83	92.19 ± 0.34	60.18 ± 39.24	63.55 ± 0.97	91.62 ± 0.66
2	93.33 ± 1.57	97.94 ± 0.54	73.93 ± 2.55	99.07 ± 0.22	96.58 ± 1.54	99.34 ± 0.39	99.74 ± 0.28	98.48 ± 0.98	99.34 ± 0.77	99.99 ± 0.01	97.84 ± 0.34
3	39.22 ± 8.46	35.78 ± 5.64	72.63 ± 3.14	29.21 ± 4.36	71.34 ± 5.66	58.82 ± 19.52	91.09 ± 2.07	0.00 ± 0.00	24.43 ± 28.69	37.47 ± 2.71	76.20 ± 3.14
4	66.64 ± 3.31	82.50 ± 1.22	92.11 ± 2.27	88.43 ± 0.41	86.14 ± 5.27	87.66 ± 2.35	98.38 ± 0.66	69.00 ± 4.96	50.62 ± 36.68	3.89 ± 1.12	89.85 ± 1.74
5	97.05 ± 4.87	97.81 ± 0.50	99.06 ± 0.23	99.24 ± 0.20	99.06 ± 0.34	99.82 ± 0.32	99.97 ± 0.08	99.13 ± 0.07	62.42 ± 45.17	5.96 ± 1.22	93.18 ± 2.27
6	41.83 ± 5.32	40.24 ± 1.71	69.81 ± 2.29	50.02 ± 3.48	74.99 ± 9.63	81.75 ± 5.51	99.21 ± 0.43	17.44 ± 0.47	29.10 ± 25.97	3.30 ± 1.37	81.54 ± 3.06
7	72.15 ± 6.70	66.55 ± 3.06	81.33 ± 3.51	50.64 ± 9.99	77.19 ± 4.18	8.00 ± 8.18	86.99 ± 6.62	0.00 ± 0.00	35.41 ± 35.18	74.58 ± 1.61	84.14 ± 1.41
8	78.98 ± 4.69	89.62 ± 1.79	65.02 ± 2.01	93.72 ± 1.35	86.71 ± 3.46	86.96 ± 11.36	94.30 ± 2.45	87.56 ± 0.95	58.91 ± 39.38	71.97 ± 0.97	88.48 ± 1.15
9	99.70 ± 0.04	99.28 ± 0.26	97.59 ± 0.64	99.87 ± 0.11	99.70 ± 0.31	96.30 ± 2.51	99.67 ± 0.34	99.54 ± 0.13	65.49 ± 44.15	75.49 ± 3.37	99.14 ± 0.26
OA	79.06 ± 0.99	83.90 ± 0.29	73.17 ± 0.95	85.86 ± 0.34	90.08 ± 0.96	90.25 ± 1.19	98.12 ± 0.26	77.08 ± 0.18	70.47 ± 17.74	66.32 ± 0.44	91.98 ± 0.49
AA	74.36 ± 1.42	77.60 ± 0.63	79.05 ± 0.51	77.87 ± 0.88	87.11 ± 1.24	79.61 ± 2.37	96.44 ± 0.75	62.59 ± 0.43	53.99 ± 28.78	48.47 ± 0.89	89.11 ± 0.84
Kappa	71.49 ± 1.33	77.92 ± 0.39	65.73 ± 1.04	80.68 ± 0.49	86.70 ± 1.36	86.86 ± 1.64	97.51 ± 0.34	67.70 ± 0.21	52.81 ± 32.51	48.31 ± 0.78	89.27 ± 0.70

**Table 12:** Wilcoxon rank-sum test results.

Dataset		KNN	RF	MLR	SVM	1DCNN	2DCNN	HybridSN	PSO-SVM	GA-SVM	ABC-SVM
Salinas	OA	9.13 × 10^−5^	9.13 × 10^−5^	9.13 × 10^−5^	9.13 × 10^−5^	9.13 × 10^−5^	5.15 × 10^−1^	1.00 × 10^0^	9.13 × 10^−5^	9.13 × 10^−5^	9.13 × 10^−5^
	AA	9.13 × 10^−5^	9.13 × 10^−5^	9.13 × 10^−5^	9.13 × 10^−5^	9.13 × 10^−5^	3.64 × 10^−3^	1.00 × 10^0^	9.13 × 10^−5^	9.13 × 10^−5^	9.13 × 10^−5^
	Kappa	9.13 × 10^−5^	9.13 × 10^−5^	9.13 × 10^−5^	9.13 × 10^−5^	9.13 × 10^−5^	5.15 × 10^−1^	1.00 × 10^0^	9.13 × 10^−5^	9.13 × 10^−5^	9.13 × 10^−5^
Trento	OA	9.08 × 10^−5^	9.08 × 10^−5^	9.08 × 10^−5^	9.08 × 10^−5^	9.08 × 10^−5^	1.36 × 10^−1^	1.00 × 10^0^	9.08 × 10^−5^	9.08 × 10^−5^	9.08 × 10^−5^
	AA	9.13 × 10^−5^	9.13 × 10^−5^	9.13 × 10^−5^	9.13 × 10^−5^	9.13 × 10^−5^	9.13 × 10^−5^	1.00 × 10^0^	9.13 × 10^−5^	9.13 × 10^−5^	9.13 × 10^−5^
	Kappa	9.13 × 10^−5^	9.13 × 10^−5^	9.13 × 10^−5^	9.13 × 10^−5^	9.13 × 10^−5^	1.21 × 10^−1^	1.00 × 10^0^	9.13 × 10^−5^	9.13 × 10^−5^	9.13 × 10^−5^
PaviaU	OA	9.13 × 10^−5^	9.13 × 10^−5^	9.13 × 10^−5^	9.13 × 10^−5^	9.13 × 10^−5^	3.84 × 10^−4^	1.00 × 10^0^	9.13 × 10^−5^	3.77 × 10^−4^	9.13 × 10^−5^
	AA	9.13 × 10^−5^	9.13 × 10^−5^	9.13 × 10^−5^	9.13 × 10^−5^	9.13 × 10^−5^	9.13 × 10^−5^	1.00 × 10^0^	9.13 × 10^−5^	8.40 × 10^−4^	9.13 × 10^−5^
	Kappa	9.13 × 10^−5^	9.13 × 10^−5^	9.13 × 10^−5^	9.13 × 10^−5^	9.13 × 10^−5^	2.91 × 10^−4^	1.00 × 10^0^	9.13 × 10^−5^	3.77 × 10^−4^	9.13 × 10^−5^
		9	9	9	9	9	5	0	9	9	9

**Table 13:** Comparison results of running time.

Runtime (min)	KNN	RF	MLR	SVM	1DCNN	2DCNN	HybridSN	PSO-SVM	GA-SVM	ABC-SVM	SFOAE-SVM
Salinas	2.15	0.16	0.12	2.62	0.69	0.93	158.37	12.04	10.94	22.32	36.80
Trento	0.20	0.11	0.12	0.27	0.35	0.55	77.70	4.87	3.82	8.36	14.36
PaviaU	1.39	0.33	0.17	0.47	0.70	1.47	151.15	8.25	6.99	13.68	19.50

## Data Availability

All data included in this study are available upon request by contacting the corresponding author.
